# Selectively expressed RNA molecules as a versatile tool for functionalized cell targeting

**DOI:** 10.1038/s41467-024-55547-6

**Published:** 2025-01-06

**Authors:** Frederik Rastfeld, Marco Hoffmann, Sylvie Krüger, Patrick Bohn, Anne-Sophie Gribling-Burrer, Laura Wagner, Nils Hersch, Carina Stegmayr, Lukas Lövenich, Sven Gerlach, Daniel Köninger, Christina Hoffmann, Helene L. Walter, Dirk Wiedermann, Hajaani Manoharan, Gereon R. Fink, Rudolf Merkel, Heribert Bohlen, Redmond P. Smyth, Maria A. Rueger, Bernd Hoffmann

**Affiliations:** 1 Institute of Biological Information Processing, IBI-2: Mechanobiology, Research Centre Juelich, Juelich, Germany; 2 Helmholtz Institute for RNA-based Infection Research, Helmholtz Centre for Infection Research, Würzburg, Germany; 3 Université de Strasbourg, CNRS, Architecture et Réactivité de l’ARN, UPR9002, Strasbourg, France; 4 Institute of Neuroscience and Medicine, INM-4: Medical Imaging Physics, Research Centre Juelich, Juelich, Germany; 5 Institute of Neuroscience and Medicine, INM-3: Cognitive Neuroscience, Research Centre Juelich, Juelich, Germany; 6 University of Cologne, Faculty of Medicine and University Hospital, Department of Neurology, Cologne, Germany; 7 Max Planck Institute for Metabolism Research, Multimodal Imaging Group, Cologne, Germany; 8 SRTD biotech GmbH, Juelich, Germany

**Keywords:** Locked nucleic acid, Drug development, RNA nanotechnology, Gene regulation

## Abstract

Targeting of diseased cells is one of the most urgently needed prerequisites for a next generation of potent pharmaceuticals. Different approaches pursued fail mainly due to a lack of specific surface markers. Developing an RNA-based methodology, we can now ensure precise cell targeting combined with selective expression of effector proteins for therapy, diagnostics or cell steering. The specific combination of the molecular properties of antisense technology and mRNA therapy with functional RNA secondary structures allowed us to develop selectively expressed RNA molecules for medical applications. These seRNAs remain inactive in non-target cells and induce translation by partial degradation only in preselected cell types of interest. Cell specificity and type of functionalization are easily adaptable based on a modular system. In proof-of-concept studies we use seRNAs as platform technology for highly selective cell targeting. We effectively treat breast tumor cell clusters in mixed cell systems and shrink early U87 glioblastoma cell clusters in the brain of male mice without detectable side effects. Our data open up potential avenues for various therapeutic applications.

## Abstract (web summary)

Targeting of diseased cells is key to the development of next-generation pharmaceuticals, but is often hindered by a lack of specific cell surface markers. Here the authors develop an RNA-based approach, which allows precise control of gene expression, with translation only occurring within preselected cell types of interest.

## Introduction

Fundamentally, all modern medical treatments against cancer, as for viral, metabolic, and genetic diseases, would massively benefit if the affected cell could be specifically identified and targeted. Such a means would allow the medication to be locally effective in the optimal functional concentrations without causing significant side effects in the surrounding tissue or the entire organism.

For decades, approaches for targeted delivery of therapeutics in cancer cells have existed and typically involve systemic administration of therapeutics packaged in nanocarriers (NCs) or localized delivery to the diseased tissue. Encapsulation of therapeutic molecules (e.g., small molecule inhibitors, chemotherapeutics, siRNA) in NCs can improve their solubility and bioavailability, alter their bio-distribution, and facilitate their entry into the target cell. “Passively” targeted NCs, which utilize the enhanced permeability and retention effect^1^ of many solid tumors, are the most extensively explored strategy for targeting cancer systemically^2^. Multiple passively targeted NCs have been approved over the past 20 years for medical applications^3^. However, due to significant uptake also by healthy cells and clearance by the mononuclear phagocytic system^4^, side effects remain high, limiting their medical use.

Active cellular targeting was developed as a complementary strategy for improving tumor localization of NCs by increasing their targeting efficiency and enhancing retention and uptake at the target site^5,6^. However, although having ultimate potential, active targeting largely depends on overexpressed or cell type-specific surface markers of diseased tissues or cells and their ability to induce internalization upon specific NC surface binding^4,7^. One of the currently most promising targeting approaches is the use of CAR-T-cells as effective medication against B-cell leukemia and others^8–10^, although the underlying targeted surface marker is not a disease-associated protein but the B-cell-specific CD19 surface protein. Furthermore, targeting approaches of just a single cell-surface receptor on tumor cells disregard tumor heterogeneity and promote selection toward the survival of resistant clones^11,12^. To overcome the need for surface markers, the latest active targeting approaches are based on well-defined lipid compositions for subsequent lipoplex formation^13–16^. Although these systems cannot distinguish between healthy and diseased cells, they allow organ- and tissue-specific targeting.

Interestingly, proteome and transcriptome analyses suppose that suitable surface markers will remain the major challenging limitation for classical active targeting approaches^17,18^. This problem is exemplified by glioblastoma brain tumors, in which several surface markers show increased expression but never true specificity^19^. The same analyses, however, revealed a much higher probability of identifying characteristic molecules on an RNA level in diseased cells, irrespective of their underlying function (e.g., mRNA, (l)ncRNA)^20–22^ and subsequent protein localization. Corroborating this idea, several glioblastoma-specific RNA molecules have been identified in liquid biopsy approaches and transcriptome analyses^23,24^. Consequently, transferring cell targeting to the RNA level within the cytoplasm would open up a formerly unprecedented range of targets and application possibilities for various types of cancer. In this direction, so-called toehold switches^25^ are able to function as inhibitors for Internal Ribosomal Entry Sites (IRES) in the absence of trigger RNAs. The presence of cell type specific modulators of toehold affinity is able to free IRES for activation. Also, target-RNA-dependent activation of adenosine deaminases for cell type-specific DNA editing to induce effector expression is a promising approach in this direction^26,27^.

Here, we develop a modular system of selectively expressed RNA molecules (seRNA) for efficient targeting and simultaneous functionalization of pre-selected cell types based on a target RNA. Using nucleic acids that can be transferred as DNA or RNA in a non-targeted manner, the resulting seRNA molecules remain inactive in healthy cells while being activated and translated in target cells only. The targeting of cells is, therefore, transferred from the outside of the cell to the immense intracellular pool of disease-specific RNA molecules. This technology is made possible by the reassembly of various highly conserved RNA-based regulatory mechanisms^28–36^. The optimal arrangement of all single-task executing domains to each other in a simple modular system guarantees translational inactivity in non-target cells. In contrast, target cells induce partial degradation of the seRNA molecules by sense-antisense interactions. As a result, degradation removes IRES-blocking sequences to enable IRES-dependent translation. Consequently, the developed seRNAs represent a valuable platform technology for cell targeting and functionalization to allow applications in biotechnology and medicine.

## Results

### A multi-element structure enables cell targeting properties of seRNA molecules

**Fig. 1:**
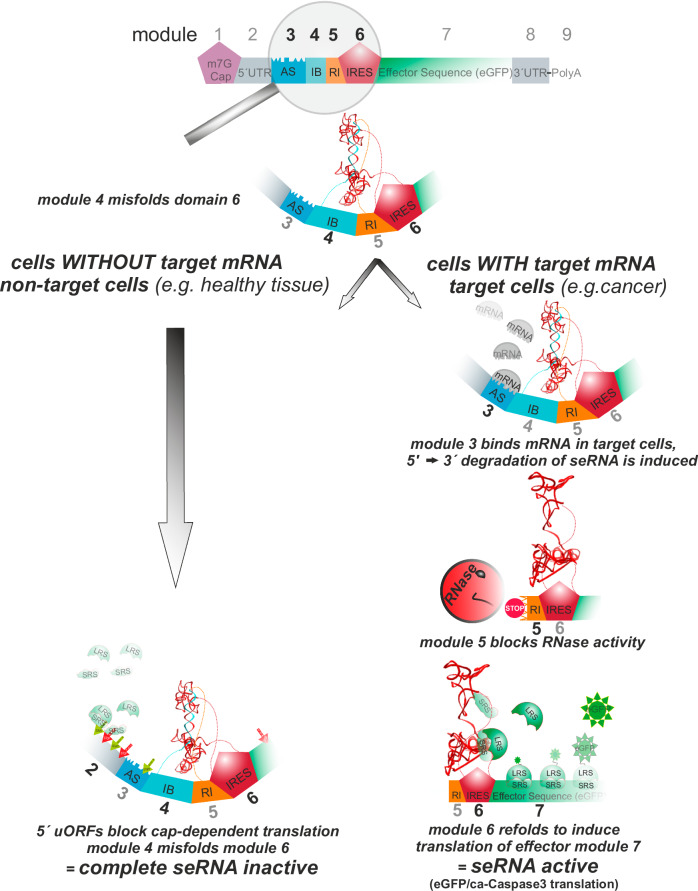
seRNA functional mechanism. Dependent on specific sense-antisense interactions, seRNA molecules induce translational activity. Upon transfer of seRNAs into non-target cells missing target mRNAs (e.g., human keratin 13, as established diagnostic marker for cancer) (left), seRNAs remain non-functional due to structural misfolding and cap-induced ribosomal silencing by uORFs (green and red arrows). In contrast, target cells (e.g., cancer cells) induce partial seRNA degradation due to sense-antisense interaction (right). Intrinsic block of degradation induces functional refolding of seRNA to allow IRES-dependent efficient translation of effector sequences in target cells only. UTR = untranslated region, AS = antisense sequence, IB = IRES blocker, RI = RNase inhibitor. Small and large ribosomal subunits are indicated as SRS and LRS.

seRNAs are comprised of multi-element molecules out of nine main functional units. The domain order is defined by an mRNA-Cap (Fig. 1, module 1), a 5’ untranslated region (5’UTR, 2), an antisense sequence (AS, 3), an IRES-blocking sequence (IB, 4), an RNase inhibiting sequence (RI, 5), an internal ribosomal entry site (IRES, 6), effector encoding sequence (7), a 3’ untranslated region (3’UTR (8), and a poly-A tail (9). While all elements are well described, an altered functionality emerges from their combination. Whilst all classical elements known from mRNAs are present (Fig. 1, module 1 + 2 as well as 7 to 9), their target-specific translational activity depends on additional sequence motifs. Most importantly, an antisense sequence (module 3) that is complementary to freely selectable target RNA is positioned as part of a 5’UTR. The 5’UTR additionally contains small upstream open reading frames (uORFs). These uORFs largely remove cap-dependent ribosomal subunits from the seRNA molecule. Such regulatory mechanisms have been known for decades and are present in various mRNAs with starvation-induced translational activation^32,36^. Further inhibition takes place by an out-of-frame AUG start codon, overlapping to the main reading frame (module 7). Since cap-dependent translation is omitted for seRNAs, an IRES (module 6) is responsible for the translational initiation of the effector ORF. However, such a construct would translate effector ORFs constitutively and independent of the cell type. We exploit structural sensitivity to IRES functionality by incorporating short RNA-sequences 5´ to the IRES that induce secondary structure rearrangements that block IRES function (IRES-blocker (IB) module 4)^35^. Having such an incomplete seRNA construct in hand, the IRES would remain inactive in non-target cell types, but a sense-antisense interaction with RNA molecules in target cells would induce unwanted degradation of the complete seRNA molecule. To protect from further degradation of downstream located seRNA sequences, we employ viral RNA sequence motifs that efficiently block 3´-directed RNA exonuclease activity^31^. By placing such a motif between the IB and IRES (module 5), seRNA molecules display their full potential with blocked IRES activity in non-target, i.e., healthy cells. In target cells, however, the formation of double-stranded RNA motifs induces seRNA degradation, removing the IRES-blocker but leaving downstream IRES and effector coding sequences unaffected to induce targeted translation and cell functionalization.

### seRNA molecules specifically target and functionalize pre-chosen cell types

**Fig. 2:**
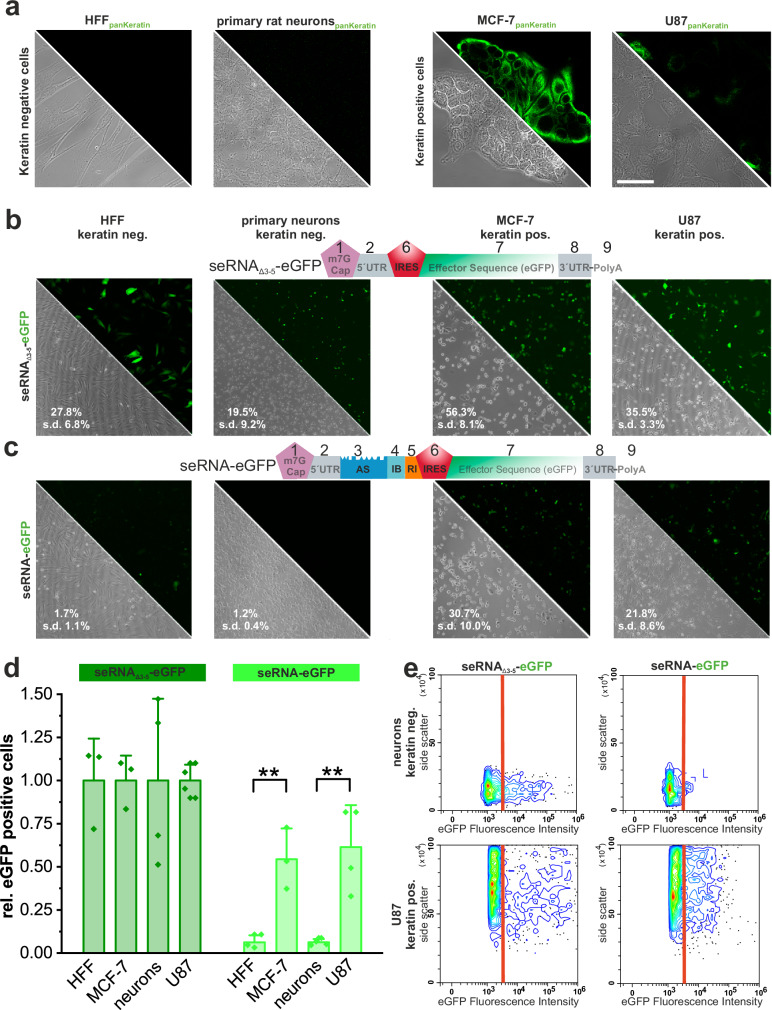
seRNA targeting specificity. Using keratin as a selectively expressed target for seRNA activation, keratin-negative human foreskin fibroblasts (HFF) and primary neurons, as well as keratin-positive breast cancer (MCF-7) and glioblastoma cell lines (exemplarily shown for SF188) were stained. Scale bar = 50 µm. **a** Non-selective and selectively expressed RNA constructs with indicated domain architecture were used for expression analysis in non-target and target cells after transfer as DNA-expression plasmid (**b**, **c**). Flow cytometry-based eGFP positive cells were either given as a percentage of all cells (**b**, **c**)) or as relative value +/− s.d. using transfection efficiencies of non-selectively expressed RNA constructs as reference. *P*-values of 0.01 based on a one-way ANOVA test are indicated by two stars (**d**). *n* = at least 3 independent experiments for (**b**–**d**). Flow-cytometry analyses of primary cortical neurons (top) and U87 glioblastoma cells (bottom) expressing non-selective and seRNA constructs. The threshold (red line) indicates 99% of all cells in an untransfected control (**e**).

To verify functionality, seRNA constructs were generated to specifically target human breast cancer and glioblastoma cancer cells using a conserved motif of the cancer diagnostic marker keratin as an antisense sequence and eGFP as the effector. The expression of keratin was confirmed by immunocytochemistry and qRT-PCR. Primary human fibroblasts and primary rat cortical neurons served as healthy non-target control cells without detectable keratin expression based on protein staining and qRT-PCR analyses (Fig. 2a and Supplementary Fig. 1).

Transfer of seRNA expression plasmids lacking regulatory targeting modules 3 to 5 (seRNA_Δ3-5_−eGFP) (Fig. 2b) led to eGFP expression in all cell types with transfection efficiencies ranging from approximately 20% for cortical neurons to 56% for MCF-7 breast cancer cells based on flow cytometry data.

In contrast, upon transfer of all elements containing seRNAs, eGFP expression was blocked almost completely in keratin-free fibroblasts and primary cortical neurons (Fig. 2c). However, the same constructs induced stable eGFP expression in about 60% of all transfected cells in the presence of the keratin target mRNA in MCF-7 and glioblastoma cells (U87) (Fig. 2c–e). Flow cytometry data additionally showed highly heterogeneous expression levels upon cap-dependent overexpression of eGFP in seRNA_Δ3-5_−eGFP constructs. In contrast, full-length seRNA-eGFP was characterized by more homogeneous expression levels in target cells (Fig. 2e). Shortening the AS domain from 625 bp to 80 bp resulted in comparable specific seRNA activation in target cells with overall slightly reduced expression intensities (Supplementary Fig. 2).

### seRNAs are activated by partial degradation of dsRNA sequences in target cells and do not induce cytokine response

**Fig. 3:**
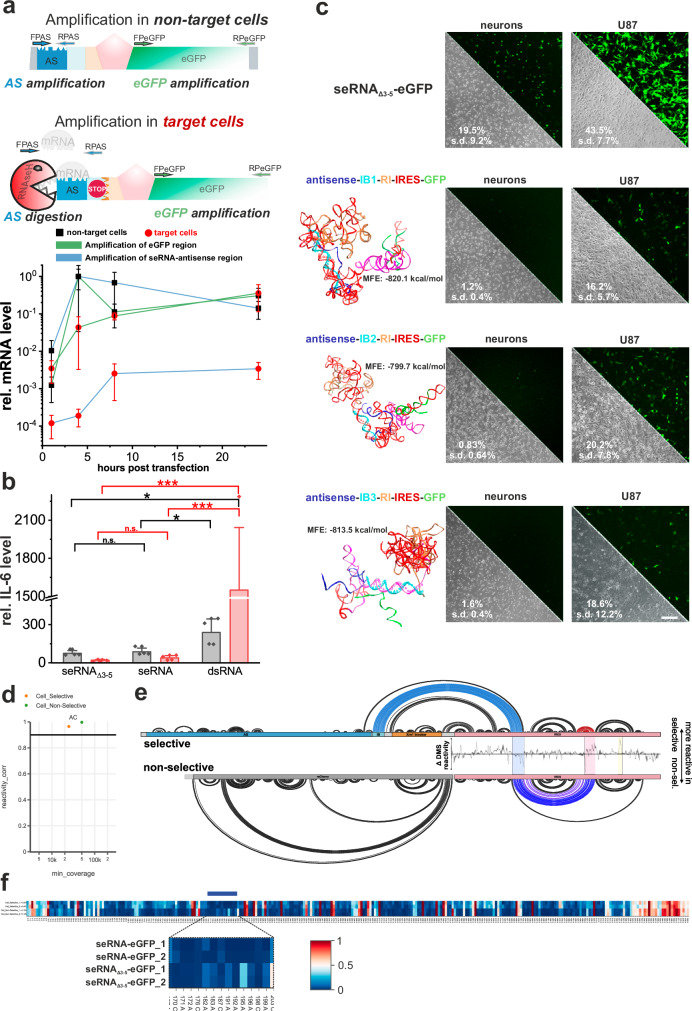
seRNA functional characterization. **a** Using two different probes in parallel, qRT-PCR experiments were performed after the transfer of seRNA-eGFP in non-target and target cells for indicated time periods. All values are given with s.d. *n* = 4 independent experiments. **b** Cytokine response analysis after transfer of stable double-stranded RNA (dsRNA), full-length (seRNA), and shortened seRNA_Δ3-5_−eGFP constructs into non-target and target cells. 24 h after transfer, IL-6 expression levels were quantified by qRT-PCR. All values are given with s.d. *n* = 4 independent experiments. Significance levels of 0.05, 0.01, and 0.001 based on a one-way ANOVA test were displayed with one to three stars. **c** Based on minimal free energy (MFE) algorithms (RNAfold WebServer), secondary structure predictions were performed for seRNA modules 4 to 6, including a few nucleotides of module 3 (antisense) and 7 (GFP) with three different IRES-blocker sequences (IB1 to IB3). For all constructs, MFE values are indicated. Their eGFP expression levels in non-target cells (neurons) and target cells (U87) are shown (green signal, right). Flow cytometry-based eGFP positive cells (in %) are indicated for IB1-3 in each image. Transfection efficiencies of seRNA_Δ3-5_−eGFP into non-target and target cells are given as control. Unaffected cell morphology is indicated in phase contrast. All values are given with s.d. Scale bar = 200 µm. *n* = 4 to 10. **d** Correlation between DMS reactivities in biological replicates (Pearson-r) and sequencing coverage (lowest number of reads). The orange dot is seRNA_Δ3-5_−eGFP, and the green dot is seRNA-eGFP. **e** Arc plot of DMS supported secondary structure predictions for the seRNA_Δ3-5_−eGFP (selective) and seRNA-eGFP (non-selective) construct with included delta DMS reactivities. Mean, black line; s.d., gray shading. **f** Heatmap of DMS reactivities at A and C residues for seRNA_Δ3-5_−eGFP and seRNA-eGFP RNA using a blue-white-red color scheme. Blue, 0; white, 0.5; red, 1.

Functional analyses of seRNA translational activation in target cells were performed for seRNA encoding expression plasmids with eGFP as an effector after transfer into the target (MCF-7) and non-target cells (HFF). Using seRNA-specific primers in simultaneous two-probe qRT-PCR analyses for sequences upstream and downstream of element 5 (RNase inhibiting sequence), stable levels of full-length seRNA molecules in non-target cells were detected over time. Specifically, seRNA concentrations increased with time to reach steady state levels after approximately 8 h (Fig. 3a), proving the expression of full-length seRNAs in non-target cells. Concentrations of seRNA 5’-regions and 3’-regions show an almost identical concentration behavior, which argues for seRNA stability. In contrast, upon seRNA transfer into target cells, qPCR analyses using upstream and downstream primers identified significantly diverging concentrations of underlying templates. While concentrations of the seRNA 3’-region increased with time to similar steady-state levels after approximately 8 h as shown for non-target cells, concentrations of the seRNA 5’ region reached steady-state levels of less than 1% of the corresponding 3’ region. These data demonstrate massively reduced lifetimes for 5’-regions upstream of module 5 and simultaneous stabilization of remaining 3’-seRNA fragments in target cells.

seRNA activation depends on the temporal intracellular RNA double-strand formation and recognition. Since double-stranded RNA molecules have been shown to induce toll-like and RIG-I-like receptor-dependent innate cytokine responses in vitro and in vivo^37,38^ with potentially massive local or systemic inflammatory responses, which conflict with medical applications, we tested for cytokine expression after transfer of seRNA expressing plasmids into target and non-target cells. Stable double-stranded RNA molecules of comparable length as antisense in seRNA molecules were taken as positive controls. Such stable dsRNAs induced IL-6 expression in non-target and target cells by a factor of up to 1350 (Fig. 3b). In contrast, no antisense-induced cytokine induction could be detected for seRNA_Δ3-5_ and full-length seRNA within 24 h (Fig. 3b). Antisense-specific IL-6 expression remained low with even lower values in target cells than non-target cultures.

We additionally tested for the robustness of IRES-blocker (IB, module 4) activity in non-target and target cells. For this purpose, we generated constructs with IB sequences complementary to either a largely unfolded IRES region (IB1), a stem-loop (IB2), or a predicted complex stem structure (IB3) based on earlier RNA structure predictions^29^. Simple secondary structure predictions based on minimal free energy calculations indicate that all IRES-blocker sequences IB1 to IB3 effectively interact with corresponding IRES modules in full-length seRNA constructs. Upon transfer in non-target primary neurons, these different IB sequences effectively inhibited eGFP expression. In U87 target cells, however, all constructs facilitated eGFP expression (Fig. 3c).

**Fig. 4:**
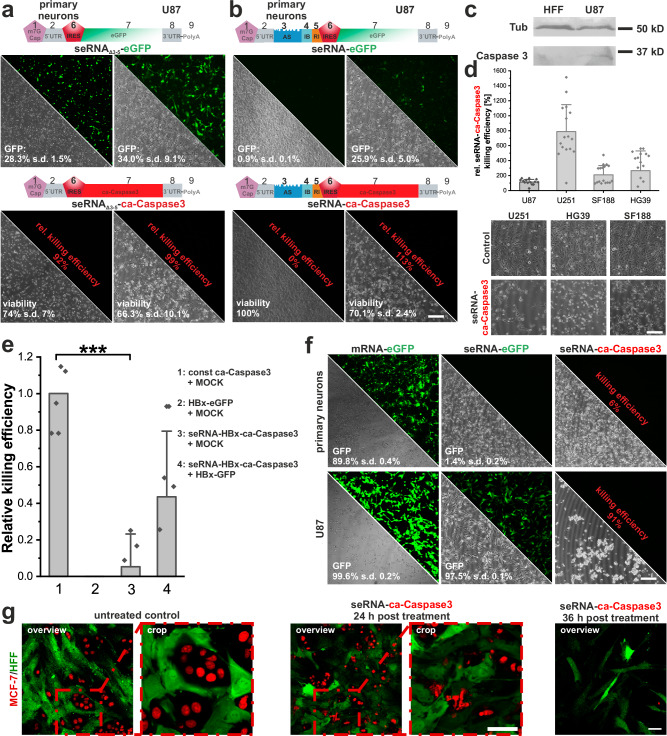
seRNA as a treatment approach against cancer and viral diseases. seRNA_Δ3-5_ (**a**) and seRNA (**b**) constructs with either eGFP or ca-Caspase3 were expressed in indicated cell types. Target-specific expression of eGFP (green) and cell viability was analyzed by light microscopy and flow cytometry, given in %. For (**a**) and (**b**) *n* = 3 or more. **c** Western blot analysis of HFF and U87 cells 24 h after transfection with seRNA-ca-Caspase3. Tubulin was used as an internal marker. *n* = 3. **d** Indicated glioblastoma cell lines were transfected with seRNA-ca-Caspase3 and tested for survival after 24 h. Toxicity is indicated in phase contrast. Numbers show relative killing efficiencies based on flow cytometry corrected by cell line-specific transfection efficiencies received with seRNA_Δ3-5_−eGFP. *n* = 10 or more independent experiments. **e** seRNA-HBx expression plasmids were co-transfected with control and HBx expression plasmids. Maximal killing efficiencies were determined by using a constitutive ca-Caspase3 expression plasmid. 24 h after transfection, killing efficiencies were determined. *n* = 4 or more independent experiments. A student’s *t* test based significance level of 0.001 is displayed with three stars. Only positive relative killing efficiencies are illustrated in the figure. **f** Switch from plasmid-based expression to IVT seRNA. Indicated in vitro transcribed seRNAs were transfected into non-target (primary neurons) and U87 glioblastoma cells and analyzed for GFP expression and cell death. As a control, an IVT GFP-mRNA was used. Absolute GFP-transfection efficiencies and killing efficiencies are indicated based on flow cytometry analysis. n = 3 independent experiments. **g** Stably red nuclear labeled MCF-7 breast cancer cells and cytoplasmic GFP labeled human fibroblasts (HFF) without seRNA treatment and treatment for indicated times with seRNA-ca-Caspase3. Nuclear shapes are magnified for indicated areas. All scale bars = 200 µm except for (**g**) with 50 µm. *n* = 3.

To further confirm our hypothesized IRES inhibition mechanism, we next measured the secondary structure of the EMCV IRES by RNA structure chemical probing with Nano-DMS-MaP^39^ in cells transfected with either the full-length seRNA-eGFP or non-selective seRNA_Δ3-5_−eGFP construct. We recovered highly reproducible structural probing data with our two biological replicates (R^2^ = 1,00 and 0,97 for seRNA_Δ3-5_−eGFP and seRNA-eGFP construct respectively). The resulting predicted secondary structure was broadly consistent with the canonical fold of the IRES. However, we could clearly detect alterations in the reactivity of the selective construct that are consistent with the interaction between the IRES-blocker and the target region within the IRES (Fig. 4d–f). This interaction perturbs the IRES structure, potentially due to steric hindrance to the formation of a pseudoknot, or altered protein interactions (Fig. 3e, f).

### seRNA-based cell targeting combines optimal specificity with effectively induced apoptosis of target cells

Due to seRNA targeting abilities with functional activation only in target cells, we replaced eGFP with a constitutively active caspase 3 (ca-Caspase3). Caspase-containing control constructs lacking the inner regulatory elements (seRNA_Δ3-5_–ca-Caspase3) proved this ability in every cell type tested. Here, approximately 90% to 100% of all transfected target and non-target cells, based on the transfer of comparable eGFP-constructs, died in the presence of ca-Caspase3 within 24 h (Fig. 4a). In contrast, full-length seRNA-ca-Caspase3 constructs were entirely inactive in non-target primary neurons while equally effective as non-selective seRNA_Δ3-5_ constructs in U87 human glioblastoma target cells (Fig. 4b). Western blot analyses confirmed selective caspase expression in target cells only 24 h after transfection (Fig. 4c). Prolonged seRNA-eGFP expression for 48 h in combination with repeated seRNA delivery further enhanced transfection efficiencies to 95% in U87 cells and ~ 70% in primary neurons. Upon use of seRNA-ca-Caspase3, the same conditions resulted in total values of 60% apoptotic cells after 48 h while primary neurons remained thoroughly unaffected (Supplementary Fig. 3). To exclude cell line-specific effects, we tested three additional glioblastoma cell lines. In all cases seRNA-ca-Caspase3 efficiently induced apoptosis. When compared to seRNA_Δ3-5_–GFP transfection rates, relative killing efficiencies were even higher than 100%, indicating the high effectiveness of ca-Caspase3 at already low protein concentrations (Fig. 4d).

The platform character of seRNAs was additionally verified by a switch from an anti-cancer to an anti-viral specificity. For this, we exchanged the cancer cell targeting keratin antisense by an antisense sequence recognizing HBx RNA of the hepatitis B virus. HeLa cells were transfected with seRNA-HBx-ca-Caspase3 in combination with either an HBx-GFP expression plasmid or an empty DNA plasmid. A constitutively active ca-Caspase3 expression plasmid was used as the positive control. seRNA activation with subsequent caspase expression and cell death induction took place only in HBx sense/seRNA-HBx antisense co-expressing cells (Fig. 4e) proving the selective functionality of the seRNA platform technology. We additionally tested the direct transfection of in vitro transcribed (IVT) full-length seRNA-ca-Caspase3 and seRNA-GFP RNA molecules in U87 target and non-target primary neurons. Compared to plasmid transfer, we observed strongly enhanced transfection efficiencies with just one single transfection. This resulted in almost all cells in bright GFP signals for seRNA-GFP and targeted killing of more than 90% of all cells upon use of seRNA-ca-Caspase3 RNA. In contrast, no seRNA activation and, therefore, the complete absence of GFP expression or induced cell death was detected in non-target cells, despite efficient transfection efficiencies as shown for control GFP-mRNAs (Fig. 4f).

Co-culture experiments with breast cancer MCF-7 target cells and equally well-transfectable human fibroblasts as non-target cells (Supplementary Fig. 4a) support targeted seRNA activation and provide clear evidence of target cell-specific apoptosis induction. Here, 24 h after transfection with IVT seRNA, seRNA-ca-Caspase3 expression induced nuclear rupture in MCF-7 cells while HFF cells largely stayed unaffected by the treatment (Fig. 4g). Quantification confirmed loss of around 70% of cancer cells from co-cultures already 24 h after transfection without any reduction of non-target cells (Supplementary Fig. 4b).

**Fig. 5:**
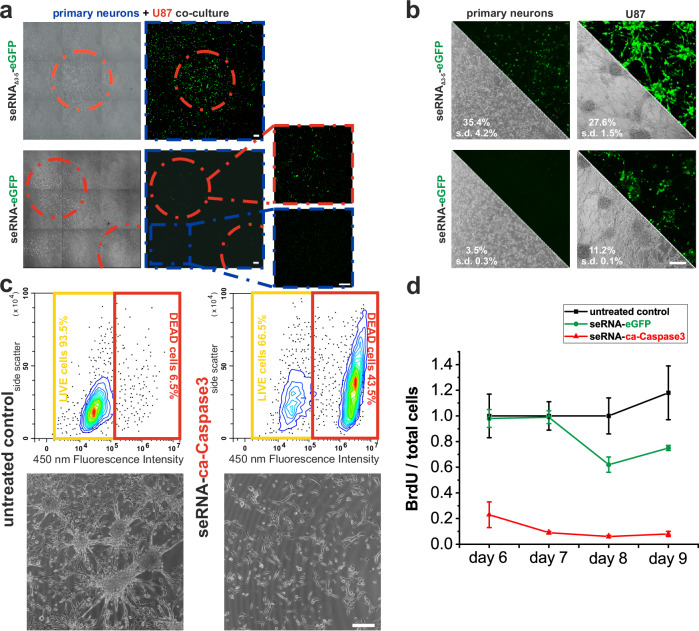
seRNAs in mixed cultures and organoids. **a** Growth of patterned U87 cell clusters (red circles) in the surrounded monolayer of primary neurons with subsequent transfer of seRNA_Δ3-5_−eGFP and seRNA-eGFP. For better visualization, zoom-in images are contrast-enhanced. **b** Transfer of the same seRNA constructs into neuronal networks and U87 tumor-spheres after growth for 5 days. *n* = 2 independent experiments. **c** Single transfer of seRNA-eGFP and seRNA-ca-Caspase3 into U87 target cells 24 h after seeding during tumor-sphere formation. Spheres are visualized in comparison to untreated U87 cells in phase contrast. Caspase activity was characterized by flow cytometry. Enhanced fluorescence at 450 nm corresponds to reduced viability. *n* = 4 independent experiments. **d** Repetitive delivery of seRNA-eGFP and seRNA-ca-Caspase3 into young U87 tumor spheres at days 2, 4, and 6 in the presence of BrdU. Subsequent cell proliferation was analyzed at indicated time points. All values are given with s.d. Scale bar = 200 µm. *n* = 2 to 3 independent experiments.

To analyze glioblastoma in co-cultures from primary neurons and U87, glioblastoma spheres were formed. A single treatment with seRNA_Δ3-5_−eGFP expression plasmids proved effective transfer into both, tumor cells and neuronal networks with subsequent non-targeted GFP expression. In contrast, the transfer of full-length seRNA-GFP confirmed also in mixed cultures the activation of effector expression only in U87 target cells and activity inhibition in non-target primary neurons (Fig. 5a). Using seRNA-ca-Caspase3 on co-cultures confirmed specific seRNA activation in all glioblastoma cell lines, while the morphology of primary neurons remained unaffected (Supplementary Fig. 5). Artificially formed solid U87 tumor spheres proved that the same target cell-specific activation of full-length seRNAs remained as functional as found on single cell level (Fig. 5b). The same tumor spheres were used to analyze the effectiveness of seRNA-ca-Caspase3 activation. While such glioblastoma-mimicking spheres formed within 5 days in the absence of seRNA-ca-Caspase3, the formation was entirely suppressed after a single seRNA transfer 24 h after cell seeding. Flow cytometry analyses confirmed induced cell death after transfer of seRNA-ca-Caspase3 (Fig. 5c). Furthermore, U87 cell proliferation was analyzed in long-term experiments using BrdU after repetitive treatment of already formed tumor spheres. Here, U87 cell proliferation remained stable on a high level in untreated cells and seRNA-eGFP expressing controls. In contrast, the transfer of seRNA-ca-Caspase3 effectively blocked proliferation by 75% to more than 90% over time (Fig. 5d).

### seRNA effectively treats early U87 glioblastoma cell clusters in mice without detectable side effects

**Fig. 6:**
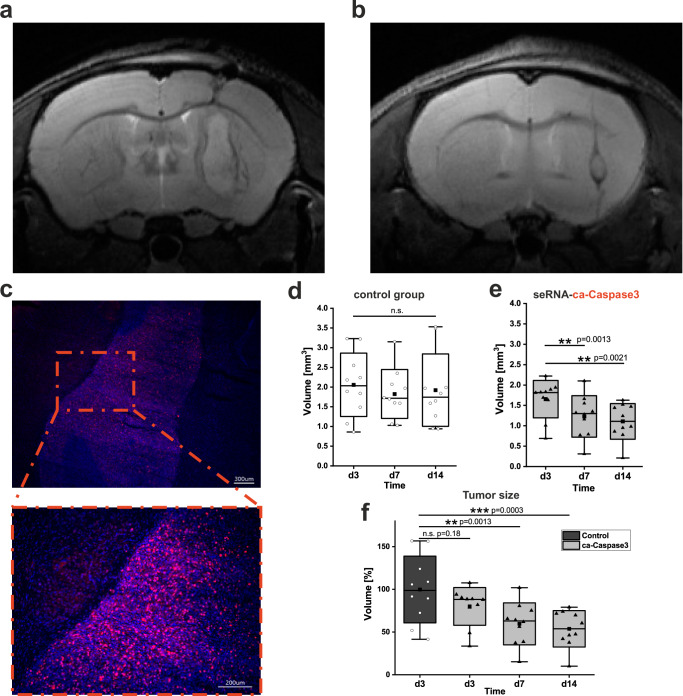
seRNA treatment of glioblastoma mice. Tumors of human U87 glioblastoma cells were grown in immunodeficient mice and analyzed by MRI 14 days after treatment with a therapeutically inactive seRNA-eGFP construct as control (**a**) or the therapeutic seRNA-ca-Caspase3 construct (**b**). Tumor formation and mitotic activity of tumor cells were verified by Ki67 immunoreactivity (red) and nuclear staining (blue) 16 days after tumor implantation (scale bar 300 µm) (**c**). A zoom-in of the red dotted square is indicated below and reveals densely packed Ki67 positive tumor cells (scale bar 200 µm). Based on MRI images, tumor volumes were evaluated after treatment with control (seRNA-eGFP or NaCl) (**d**) or therapeutic seRNA-ca-Caspase3 constructs (**e**) at indicated time points. Statistical analyses were performed with repeated measures of one-way ANOVA over the factor time with *p* = 0.5035 for (**d**) and *p* = 0.0001 for (**e**). For direct comparison of control and therapeutic animals, relative tumor volumes in relation to day 3 (d3) control mice are shown. Statistical analysis was performed via one-way ANOVA over the factor time (**f**). For all statistical analyses box plots provide all single values for every time point. Mean values are given as the center point, the median as a line with whisker min/max values, and the s.d. as a box. Significance levels of 0.05, 0.01, and 0.001 were displayed with one to three stars. For (**d**–**f**) *n* = 10 for each group and time point out of 4 independent experiments.

seRNA functionality was further verified in an in vivo proof of concept analysis in immunodeficient mice. Human U87 glioblastoma cells were injected into the striatum and grown for two days to allow for robust and mitotically active brain tumor formation (Fig. 6a, c). Treatments in independent control groups with either isotonic NaCl solution or full-length seRNA-GFP expression plasmids by stereotactic injection directly into the tumor showed highly comparable results without affecting tumor growth over time. In contrast, injection of the seRNA-ca-Caspase3 construct into glioblastoma visibly reduced the tumor size as assessed by non-invasive MRI 14 days after treatment (Fig. 6b). Monitoring tumor development over time by MRI revealed continuous tumor shrinkage induced by caspase-expressing seRNA (Fig. 6e) as compared to tumors treated with control constructs (Fig. 6d*p* = 0.6231). Likewise, sizes of caspase-seRNA treated tumors were significantly reduced in relation to control tumor volumes on day three (Fig. 6f). Detailed daily monitoring showed no cytotoxic effects or behavioral changes in the treated animals at any time point.

**Fig. 7:**
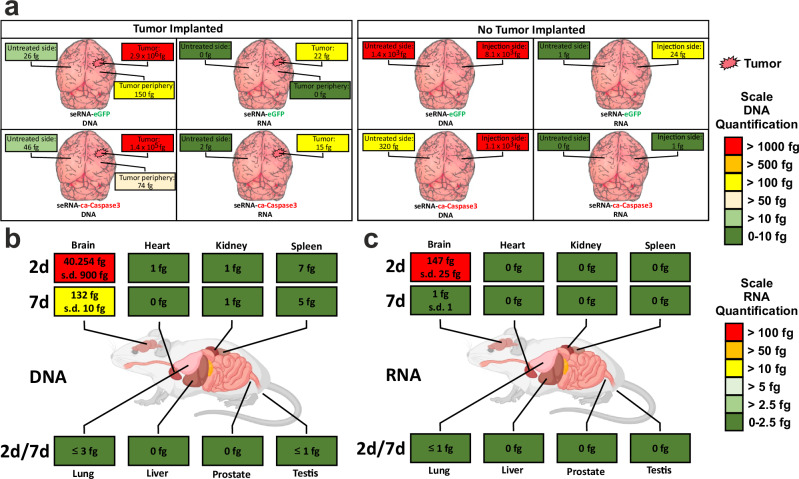
seRNA in vivo diffusion analysis. **a** Mice with or without early U87 tumors (day 3) were treated with seRNA plasmids expressing either GFP (top) or ca-Caspase3 (bottom). After an additional two days, mice were sacrificed, and brain tissue samples from indicated positions were taken and tested for seRNA encoding plasmid DNA and expressed seRNA. Figure shows data from single mice, taken from a series of experiments. Same qPCR (DNA) (**b**) and qRT-PCR (RNA) (**c**) analysis were performed for indicated organs 2 as well as 7 days after seRNA-ca-Caspase3 brain transfer. For each time point 4 treated and 2 untreated mice were analyzed. Values are given as mean. For the brain as the only organ with detectable seRNA, values are given with s.d. Created with BioRender.com.

For diffusion tests, brain tissue samples of glioblastoma mice and control animals without tumors were analyzed for seRNA-encoding plasmid DNA as well as seRNA expression 24 h after treatment. Intratumoral injection resulted in high DNA levels and effective expression in most animals tested independently on the chosen seRNA construct. Concentration dropped in the tumor periphery and was almost absent in tissue samples of the untreated brain hemisphere (Fig. 7a). In contrast, the distribution of seRNA-encoding plasmid DNA was clearly enhanced in tumor-lacking brains. Here, high DNA concentrations were found not only at sites of injection but also in tissue of the other hemisphere. RNA levels in tissue samples of tumor-lacking animals also demonstrated efficient uptake and expression. However, at no time, seRNA treatment induced any kind of toxicity or impaired animal behavior.

Since effective diffusion of seRNA-lipoplex took place, we additionally tested for plasmid-based seRNA-lipoplex crossing of the blood-brain barrier with subsequent organ accumulation and seRNA activation. While high levels of DNA (Fig. 7b) and expressed RNA (Fig. 7c) were detected in the brain 2 days after treatment, neither seRNA-based DNA nor RNA was detectable in any other organ tested. Also prolonged incubation times did not result in seRNA-based nucleic acid accumulation, and only minor DNA amounts in the brain were still detectable for prolonged times.

## Discussion

Techniques for efficient and freely adaptable cell targeting constitute one of the most urgent needs in biotechnology and medicine. Many different approaches have been developed, most of them depending on cell-type-specific surface markers^40,41^, which are, unfortunately, most often missing. With seRNAs we have developed a platform technology that allows cell targeting on the RNA-expression level by shifting recognition from the outside to the inside of the cell and by combining this type of targeting with cell type-specific translation of freely selectable effector proteins.

Current seRNA molecules are based entirely on natural sequence motifs to combine their properties into an altered mode of functionality. They bring together cell-type specificity of antisense RNA with cell functionalization abilities of mRNA techniques. This is made possible by regulated partial degradation in target cells only, which results in translational induction at IRES-dependent secondary sites. The encephalomyocarditis virus (EMCV)-IRES used here for this purpose is comprising a homogeneous translation efficiency with functional independence from viral cofactors^29^. EMCV-IRES-based expression rate is typically lower than cap-induced translation^42^ which is in line with our expression data for seRNAs. We assume that EMCV-IRES can be easily replaced by other IRES motifs to thus add further IRES-specific functionalities to seRNA molecules, such as different expression rates or cell type-specific regulation by binding proteins^43^.

The efficient blocking of IRES activity in full-length seRNA is crucial for its selective expression activity, especially for possible medical approaches, as suggested here with our proof-of-concept study against glioblastoma with vulnerable healthy brain tissue in direct contact with the tumor. Although knowledge about the exact functional mechanism of IRES molecules is still incomplete, recent literature indicates that IRES secondary structures are rather flexible and can induce cap-independent translation precisely because of this flexibility^44^. The IB motifs we use represent complementary sequence motifs to IRES sequences, which presumably block IRES functionality by disrupting RNA structure through intra- or inter-molecular interactions, as supported by Nano-DMS-MaP RNA structure probing experiments. This mechanism is similar to already described toehold motifs^25^. However, while toehold sequences represent stable sequence motifs that function via finely tuned affinity behavior to IRES sequences and cell type-specific RNA molecules, IB sequence motifs of seRNAs just interact with the IRES. Furthermore, our results on different IBs suggest a rather robust function without major differences for chosen IRES-IB interaction sites.

To allow cell-type-specific activation, interactions between the seRNA antisense and cell-type-specific RNA must occur. Sense-antisense interactions are a common, natural regulatory mechanism that acts at different levels and can regulate both the transcription itself and the amount of free sense-RNA molecules^30,45^. Upon formation, RNA-RNA double-strands are preferentially recognized by RNase III family members^46^ which, unfortunately, cannot be properly blocked for detailed analysis. However, although with a 25-fold preference for RNA/DNA hybrids^47^, RNase H1 can also bind and digest to some extent RNA/RNA double-strand duplexes^48,49^. This induces complete Xrn1-dependent degradation of RNA^50,51^. For seRNA molecules we could demonstrate a minor but significant influence of RNase H on recognition and primary cleavage (Supplementary Fig. 6). Further analysis will show to which length antisense sequences can be shortened in seRNAs and whether mismatches are tolerated. Analyses for RNase H and RNase III indicate that even shorter antisense sequences than the minimal length of 80 nucleotides used here should be sufficient for seRNA activation^52,53^. Furthermore, seRNA activation seemed to be robust against the expression intensity of sense-RNA targets since used keratin-positive glioblastoma cell lines varied clearly in detectable keratin levels (Supplementary Fig. 1) but showed effective seRNA activation in all cases.

RNase-induced complete degradation of seRNA molecules upon activation in target cells is inhibited by RNA sequence domains of the dengue virus. These sequences form specific secondary structures to inhibit further degradation by presumably altering the tertiary structure of the Xrn1 exonuclease to naturally induce the formation of a disease-related flaviviral subgenomic RNA^54,55^. The identical function of this sequence is also conserved in seRNA molecules and thus allows degradation of the 5’-upstream sequences including the IB, but stabilizes the IRES and effector sequences for cell-type-specific activation of translation.

In non-target cells, seRNA remains inactive. Besides using IRES-blocker sequences, cap-dependent translational initiation also had to be blocked for full-length seRNAs when encoded on expression plasmids. We have achieved this by incorporating upstream open reading frames (uORFs) in domains 2 to 6. The general mode of action of uORFs was already identified more than 35 years ago for yeast GCN4 mRNA^32^ and is also demonstrated for animal transcripts^56^. Depending on the surrounding sequence of a stop codon, the length of the uORF itself as well as the distance between the reading frames, they regulate not only translation initiation at the start codon of the next ORF but also partial loss of ribosomal subunits from the mRNA itself^28,57^. For our seRNA constructs, up to 6 uORFs were used. Interestingly, seRNA molecules can be delivered also as IVT RNA. This results in enhanced transfection efficiencies while keeping target cell-specific effector expression unaffected. Apoptosis-induced loss of breast cancer cells in co-culture with human fibroblasts (Fig. 4g) argues for the fast and robust efficacy of IVT seRNA. Upon the use of such IVT seRNAs, modification of the 5’ cap to impair ribosomal binding might further simplify future seRNA molecules.

Innate immune responses can largely hamper the effectiveness of various medications. Especially in viral systems, pathogen-associated molecular patterns represent optimal recognition motives for intracellular and extracellular pattern recognition receptors (PRRs). Thereby, particular viral sequence motifs, secondary structures, and especially dsRNAs are efficiently recognized mainly by toll-like and RIG1-like receptors and converted into an antiviral immune response^58,59^. Based on no detectable IL-6 expression, our data indicate that intracellularly formed seRNAs do not induce an immune response, no matter if sense-antisense interaction is taking place in target cells or not.

For proof of biotechnological and medical applicability, we equipped our seRNAs with a continuously active form of caspase3^60^ as an effector and tested targeted expression in U87 glioblastoma cells in vitro and in a simple in vivo mouse glioblastoma model. Different treatment approaches have been developed to enter preclinical and clinical phases including siRNA, mRNA, and small molecules^61,62^. All approaches failed for different reasons, and for the moment we also cannot say if the seRNA platform technology will open perspectives in glioblastoma treatment. The first proof of concept data presented here are very promising but further in vivo analysis will be necessary. In particular, the treatment of alternative glioblastoma animal models will be important^63^, as the U87 glioblastoma model used here forms sharp tumor boundaries with a resulting non-invasive growth behavior^64^. Further studies on the treatment of fully established tumors, as well as repeated application combined with an extended observation period will additionally help to verify the potential of seRNA-based therapeutics. However, our seRNA approach confirms that intracellular molecules can no longer be used only as diagnostic but also as therapeutic markers to facilitate the targeted expression of therapeutically highly active proteins exclusively in target cells. The use of antisense against keratin certainly limits seRNA delivery to intratumoral injection for glioblastoma. Other administration routes, e.g., intravenous injection, would depend on glioblastoma-specific RNAs^23,24^ and also on further advanced RNA delivery systems for passing the blood-brain barrier. Parallel scoring of glioblastoma mice during treatment did not detect any behavioral or neuronal deficits, supporting the high biocompatibility of seRNAs. Due to the simple modular design of seRNAs, we were also able to shift target specificity from cancer to cells expressing viral RNAs, underscoring the potential for diverse medical applications in the fields of oncology, virology, or gene therapy.

## Methods

### Ethical statement

The State Agency for Nature, Environment and Consumer Protection (Landesamt für Natur, Umwelt und Verbraucherschutz North Rhine-Westphalia; number 81-02.04.2019.A396) approved all animal experiments, which were conducted under the national Law for Animal Protection and European regulations and guidelines. Nude male immunodeficient mice (MRI-Foxn1nu/nu; Charles River Laboratories, France) were housed in individually ventilated cages in a specific pathogen-free environment (Tecniplast, Blueline). Food and water were sterilized and provided ad libitum. Starting with tumor cell implantation until the end of each experiment, careful scoring of the animals was performed on a daily basis using the Body Condition Scoring (BCS) system^65^. Even mild changes in behavior and neurological functions were defined as termination criteria during the experiments to ensure minimal stress for the animals. This maximal burden was not exceeded at any time.

### seRNA construction

pmCherryC1 (Addgene) was used as a backbone plasmid for seRNA construction. The human keratin 13 antisense fragment was amplified from plasmid HK13deltaT.eGFP (provided by Reinhard Windoffer, Aachen) with primers P1 and P2 (see Supplementary Table 1). IRES blockers were implemented with primers P3 and P4 (IB1), P5 and P4 (IB2), and P6 and P4 (IB3). RNase inhibitor region was generated according to the Eurofins Genomics DNA RNA oligonucleotide synthesis protocol with primers P7 and P8 (part 1) and P9 and P10 (part 2) (see Supplementary Table 1). EMCV-IRES was isolated from pFR_wt (Prof. Palmenberg WI, USA) with primers P11 and P12. The constitutively active version of caspase 3 was published before^60^ and cloned accordingly. All primer sequences, including restriction sites used for cloning, are indicated in Supplementary Table 1. Primers were ordered from Eurofins Genomics. For HBV constructs, the keratin 13 antisense sequence was replaced by an HBx antisense (nucleotides 1424 to 2053 of the HBV genome) using enzyme digestion. The antisense sequence was ordered from Eurofins Genomics. The lengths of all seRNA domains are indicated in Supplementary Table 2).

### RNA secondary and 3-dimensional structure prediction

Effects of IB1-IB3 on seRNA secondary structure were predicted using the RNAfold WebServer application (ViennaRNA package, version 2.4.13^66^). Secondary structure predictions were based on minimum free energy (MFE) and partition function. Isolated base pairs were avoided. For 3D RNA structure prediction, 3dRNA v2.0 was used in optimization and slow assembly mode. Predicted 3D structures were transferred in dot-bracket format to the Chimera 1.14 software for 3D animations, image generation, and 3D structure superimposition.

In situ, Nano-DMS-MaP-guided structures were predicted as described before^67^. Briefly, the DMS reactivity of A and C residues was averaged between both replicates before performing data-guided prediction with Eternafold v1.3.1. The predicted structures were subsequently visualized in Varna v3.93. The difference in average DMS reactivity per position between the selective and non-selective transcript for the shared IRES sequence was calculated with a custom Python script and integrated into the structure visualization.

### Cell culture

All cell types were cultivated at 37 °C in a humidified atmosphere containing 5% CO_2_. Cell morphologies as indicated in Supplementary Fig. 7 were verified throughout. Human foreskin fibroblasts (HFF) ((SCRC-1041, ATCC), 86HG39 glioblastoma cells (kindly provided by IBG-1, Research Center Juelich, Germany), HeLa cells (CCL-2, ATCC) and HEK293T cells (CRL-3216, ATCC) were cultivated in DMEM Glutamax medium (Gibco) supplemented with 10% FBS (Gibco) + 1x PenStrep (Thermo Fisher). To cultivate the breast cancer cell line MCF-7 (HTB-22, ATCC), RPMI 1640 GlutaMax medium (Gibco) was used. The medium was supplemented with 10% FBS, 1x PenStrep, 1x NEAA MEM (Sigma Aldrich), 1x sodium pyruvate (Thermo Fisher), and 10 µg/ml insulin (Sigma Aldrich). Human U87 MG glioblastoma cells (HTB-14, ATCC) and U-251 MG glioblastoma cells (9063001, Sigma Aldrich) were cultivated in MEM medium supplemented with 10% FBS, 1x PenStrep, 1x NEAA MEM, and 1x L-glutamine (Thermo Fisher). Cultivation of SF188 human glioblastoma cells (SCC282, Sigma Aldrich) were performed in MEM medium supplemented with 10% FBS, 1x PenStrep, and 1x L-glutamine (Thermo Fisher). For co-culture experiments of U87 and primary cortical neurons and U87 sphere formation, a 1:1:1 mixture of RPMI GlutaMax medium (supplemented with 10% FBS and 1x L-glutamine), U87MG medium and primary cortical neuron medium was used. For co-culture experiments of HFF and MCF-7, we used a 1:1 mixture of their respective media. Cells were seeded 24 h before plasmid transfer on 24-well plates (BD Falcon; Thermo Scientific, USA) for microscopy, flow cytometry, and qRT analysis. Primary cortical neurons from Wistar rats were isolated as described previously^68^. Briefly, cortices were prepared in ice-cold Hanks Balanced Salt Solution without Mg^2+^ or Ca^2+^ (HBSS- Gibco). The tissue was dissociated by pipetting. Then, two volumes of neurobasal medium (NB, Invitrogen) were added, and the cell suspension was incubated for 3 min. The top half of the suspension was transferred and centrifuged at 200 × *g* for 3 min, and cells were resuspended in supplemented NB medium (50 mL NeuroBasal, 2% B-27 (Gibco), 1% Glutamax, 0,1% gentamicin). Cells were plated on coated surfaces. For Nano-DMS-MaP HEK293 cells were seeded 24 h before transfection on 6-well plates (BD Falcon; Thermo Scientific, USA).

### Transfection (in vitro)

For seRNA encoding plasmid transfer, Lipofectamine 3000 was used with 1 µg plasmid DNA. Transfection was stopped after 4 h by replacing with a fresh medium. Primary cortical neurons were treated by nucleofection using the Amaxa nucleofector kit for neurons and the Amaxa program G-013. Human foreskin fibroblasts were treated with an Amaxa nucleofector kit [Kit NHDF, Amaxa program U-020 NHDF human neonatal] with 2 × 10^6^ cells and 2 µg plasmid DNA.

For HBx co-transfection experiments, the same amounts of plasmids were mixed. 1 µg plasmid DNA was delivered with Lipofectamine 3000. As nonsense (MOCK) plasmid DNA, an empty mammalian expression vector was used.

For Nano-DMS-MaP, 1 × 10^6^ cells were transfected with 600 ng of plasmid DNA, 100 µL OptiMEM (Gibco), and 7,2 µL of 1 mg/mL transfection grade linear polyethylenimine (PEI MAX MW 40,000; Polysciences), following the manufacturer’s recommendations.

### DMS probing and RNA extraction

24 h post-transfection, DMS probing was carried out by adding drop by drop 500 µL of fresh media containing 100 mM or 200 mM Dimethyl Sulfate (DMS, Sigma); final concentrations in the 2.5 mL media were 20 mM and 40 mM DMS. Cells were placed back in the incubator for 6 min (37 °C) and then transferred on ice. The media is immediately removed, and the reaction was quenched by carefully washing the cells with 1 mL of cold DPBS (Gibco) containing 10 µL β-mercaptoethanol (14.3 M, Roth). For each sample, equivalent control reactions were performed, incubating cells for 6 min at 37 °C in the presence of 500 µL of fresh media in the absence of DMS. DMS-treated and untreated control RNA samples were performed in parallel. Total cellular RNA was purified by extraction with Tri reagent LS (Sigma), according to the manufacturer’s instructions. Purified RNAs were resuspended in RNase-free H_2_O. Then, 10 µg of total cellular RNA was treated with 1.5 µl Turbo DNase (ThermoFisher Scientific), 20 U of RNasin, and 10 µl of 10 × Turbo DNase buffer in a 100 µl volume for 30 min at 37 °C. Following DNase treatment, RNA was column-purified using NTC buffer and the NucleoSpin Gel and PCR Clean-up kit (Macherey-Nagel), according to the manufacturer’s instructions

### Reverse transcription

For Nano-DMS-MaP, reverse transcription was performed on cellular RNA using MarathonRT^29^ and the RT-primer Universal_Rv AACAGCTCCTCGCCCTTGCTC. 1 µg of RNA was mixed with 0.96 mM dNTPs and 577 nM of primer brought to 13 µl with RNase-free H_2_O and denatured for 5 min at 65 °C. Samples were placed on ice for 2 min, and reverse transcription was initiated by adding 80 U MarathonRT, 8 U RNasin, in 50 mM Tris-HCl pH 8.5, 200 mM KCl, 20% glycerol (v/v), 1 mM MnCl_2_, 5 mM DTT in a 25 µl total volume. Samples were incubated for 6 h at 42 °C. cDNAs were column-purified using NTC buffer and the NucleoSpin Gel and PCR Clean-up kit (Macherey-Nagel), according to the manufacturer’s instructions, and eluted in 20 µL NE buffer.

### PCR amplification of seRNA

seRNA-eGFP samples were amplified using primer pairs Selective_Fw (GATGTCGGCCTCCACGCTC) and Universal_Rv (AACAGCTCCTCGCCCTTGCTC); seRNA_Δ3-5_−eGFP samples were amplified using Non-Selective_Fw (CGGTCGCCACCATGGTGAG) and Universal_Rv. PCR amplification conditions were 10 µl of purified cDNAs with 0.05 U of PrimeSTAR GXL polymerase (Takara Bioscience), 250 nM of each primer, 200 µM of each dNTP, and 1 × PrimerSTAR GXL buffer in a total volume of 50 µl. Cycling conditions were: initial denaturation for 2 min at 98 °C, followed by 25 cycles of 10 s at 98 °C, 15 s at 55 °C and 1 min at 68 °C, followed by a final extension for 7 min at 68 °C. Amplicon quality was checked on 1 % agarose gel poststained in EtBr.

### Nanopore sequencing

DNA amplicons were purified via SPRI bead (Mag-Bind® TotalPure NGS, Omega Bio-Tek) purification by the addition of 0.7 × volumes of beads. Next, 80 ng of DNA from each sample was prepared for Nanopore Native Ligation Barcoding Sequencing (SQK-NBD114-96, Oxford Nanopore Technologies) as described^69^. After library preparation, 20 fmol of the library was loaded onto a R10.4.1 flow cell (FLO-MIN114, Oxford Nanopore Technologies) on a Minion Mk1B sequencer (Oxford Nanopore Technologies) and sequenced for 20 h using MinKnow acquisition software (Oxford Nanopore Technologies) v. 23.07.8.

### Basecalling and isoform detection

The acquired raw data was basecalled post-run with dorado v.0.4.1 (Oxford Nanopore Technologies) with the model ‘dna_r10.4.1_e8.2_400bps_sup@v4.2.0’ and kit-name ‘SQK-NBD114-96’ followed by demultiplexing of the generated bam file with ‘dorado demux’.

Reads were then aligned to their specific reference sequences using LAST v.1419, by first indexing the transcript reference with ‘lastdb –uNEAR –R01’, then training mismatch matrices per sample with ‘last-train –Q0’, followed by alignment with ‘lastal -Qkeep –m20 –p {mismatch_matrix_file} | last-split –m1.’ The output maf file was then converted to a Sam file with the ‘maf-convert sam’ command. The SAM file was then processed using Samtools v.1.12. Briefly, using Samtools a header was added, the file was converted to BAM format, followed by sorting the BAM file and lastly indexing it. The final BAM files were then used as input for the mutational profiling analysis. Mismatch types were analyzed from BAM alignment files with perbase v.0.8.3 and custom Python scripts.

### Mutational profiling analysis

Mutational Profiling analysis was performed using RNAFramework v.2.8.6 (ref. ^24^). Mutation counting was performed with rfcount (‘-mf mask_primers.csv –-only-mut ‘G > Y;A > B;C > D;T > V’ –eq 10 –q 16 –mm –nd -ni’) followed by reactivity normalization with rf-norm (‘–scoreMethod Siegfried –normMethod 2 –reactiveBases AC –maxUntreatedMut 0.2 –maxMutationRate 0.8 –norm-independent’). Calculating the correlation of reactivity between the two replicates was performed per sample and transcript with the RNAFramework tool rf-correlate. The reactivity of biological replicates were combined for plotting and folding prediction via the rf-combine tool. Figures were generated using the Python library plotly v.4.14.3.

### Western blot analysis

U87 target and HFF non-target cells were cultured in 6-well plates (Greiner) and transfected with selective ca-Caspase3 plasmid. After washing with cold 1x PBS, cells were lysed in 100 µl RIPA lysis buffer (Sigma Aldrich) supplemented with 1% protease inhibitor (Sigma Aldrich). Lysates were centrifuged, supernatant were mixed with 5x loading dye (Bio-Rad), heated for 5 min at 95 °C, and loaded onto a 4%-20% gradient gel. Proteins were separated for 2,5 h at 100 V and then blotted to a nitrocellulose membrane (Thermo Fisher). We used Anti-Tubulin Y1/2 clone 1:500 (MAB 1864, Millipore) as a constitutively expressed marker and Anti-cleaved-Casp3 (Asp175) (Sigma Aldrich) 1:1000 to quantify the expressed caspase. Secondary antibodies Anti-Rat (A8438, Sigma Aldrich) and Anti-Rabbit (A3687, Sigma Aldrich) were both coupled to alkaline phosphatase and visualized by use of BCIP/NBT-blue liquid substrate system (Sigma Aldrich).

### Coculture experiments

To cultivate primary cortical neurons and U87 glioblastoma cells, silicone rubber inserts with 1 mm diameter circular cut-outs were produced. U87 cells were seeded in these holes, cultivated overnight, and transfected with Lipofectamine 3000 (Thermo Fisher) as indicated above. Subsequently, PDMS inserts were removed, and primary cortical neurons were seeded on top. These cells were previously treated by nucleofection to transfer corresponding seRNA or control constructs.

For co-culture of breast cancer cells with human foreskin fibroblasts, stably expressing NLS-RFP MCF-7 cells and stably expressing GFP HFF cells were used. For culturing, 90 000 HFF cells were seeded on a fibronectin-coated glass substrate (µ-Dish 35 mm, high, Ibidi). After 24 h 145 000 MCF-7 cells were added. Cells were transfected after an additional 24 h with 8 µg seRNA-Caspase3 RNA using 8 µL Lipofectamine 3000 in 1 mL media.

### U87 sphere formation

To induce sphere formation, U87 cells were seeded with 200 000 cells / 24 well plates (BD Falcon) in triple medium as indicated above. Cells were transfected with seRNA plasmids either 24 h (effect on tumor formation) or 5 days after seeding when spheres were fully developed.

### In vitro RNA synthesis

seRNA-GFP and seRNA-ca-Caspase3 coding sequences were cloned into pBluescript SK-vector. Plasmids were linearized and used as templates for T3 primer driven in vitro RNA synthesis using mMESSAGE mMACHINE T3 High Yield capped RNA Transcription Kit (Thermo Fisher, USA) IVT RNA was purified by Phenol:chloroform extraction and isopropanol precipitation using TRIzol Reagent (Thermo Fisher, USA). As positive RNA transfection control an IVT eGFP-mRNA was used. This mRNA was transcribed from the pSTIA120 pDNA, described here^70^. The RNAs were transfected using Lipofectamine 3000, just without the use of P3000 reagent.

### Immunocytochemistry

For immunohistochemistry, cells were cultivated on 35 mm glass bottom µ-dishes (Ibidi GmbH, Germany) After cultivation for 24 h, cells were washed twice with PBS (2 ml prewarmed). Afterward, cells were fixed with 500 µl ice-cold 100% methanol for 20 min at − 20 °C. Then cells were washed with 2 ml PBS at room temperature (RT) on a 2D shaker for 5 min. All samples were blocked with 500 µl blocking buffer (PBS supplemented with 5% normal goat serum and 0.3% Triton X-100, (v/v)) for 1 hour at RT on a 2D shaker. Pan-Keratin antibody (Mouse mAb conjugated with Alexa Fluor® 488, C11, mAB#4545) (Cell Signaling Technology, USA) was added 1:100 in antibody dilution buffer (PBS supplemented with 1% BSA and 0.3% TritonTM X-100, (v/v)) and incubated overnight on a 2D shaker at 4 °C. Subsequently, all samples were washed three times with 1 ml PBS for 5 minutes at RT on a 2D shaker. Samples were then either imaged directly or stored in PBS at 4 °C.

### Flow Cytometry

Flow Cytometry (CytoFLEX S Flow Cytometer, Beckmann Colter) determined eGFP-mRNA transfer efficiency and fluorescence intensity. Briefly, 24 h after transfection, the cells were trypsinized (0.05% trypsin-EDTA solution, Sigma Aldrich, USA) and centrifuged at 300 × *g*. Without fixation, cells were analyzed directly after resuspension in 250 µl of the corresponding culture medium. At least 10,000 cells were analyzed for granularity and size by forward scattering for each cell type. eGFP fluorescence intensities were measured using appropriate filter settings and gatings. Supplementary Fig. 8 exemplifies the gating strategy.

### Live-dead staining

Cell viability was analyzed by flow cytometry according to the manufacturer protocols using the LIVE/DEAD™ Fixable Red Dead cell stain kit (L34971) and violet Dead cell stain kit (L34950, Thermo Fisher) with appropriate filter settings. Cells in the supernatant were included by centrifugation also to include already detached cells. Killing efficiencies were calculated by the percentage of living cells 24 h after seRNA-Caspase3 RNA treatment relative to the number of cells after seRNA-eGFP RNA treatment.

### Cell proliferation analysis

Cell proliferation in U87 spheroids was analyzed following the product protocol using the CyQuant NF Proliferation Assay (Thermo Scientific). Fluorescence intensities were quantified using a plate reader (TECAN Austria GmbH, infinite M1000 PRO).

### Microscopy

Live cell analyses were performed in phase contrast and fluorescence 24 h after plasmid transfer at 37 °C and 5% CO_2_ using a confocal laser scanning microscope (cLSM 710, Carl Zeiss Jena, Germany). The microscope was equipped with a 488 nm argon laser and appropriate filter settings to visualize eGFP and AlexaFluor488. All images of transfected cells showed a representative overview at the center of each substrate and were recorded with an EC Plan-Neofluar 10x/0.3 Ph1 objective (Carl Zeiss Jena). Immunofluorescence and MCF-7/HFF coculture images were recorded with an EC Plan-Neofluar 40x/1.30 Oil Ph3 objective (Carl Zeiss Jena). For all experiments, microscope settings were kept unchanged. Scale bars for vector graphics were generated using the ImageJ open-source plugin Quick Figures, Version 2023.2. For fluorescence intensity measurements and cell coverage analyses of fluorescently labeled co-cultures, Python-based custom codes have been developed and are provided in the Supplementary Information as Supplementary Codes 1 and  2.

### q(RT)-PCR experiments

For qRT-PCR analyses, total RNA was isolated with the RNeasy Plus Mini kit (QIAGEN) at different time points after transfection. cDNA synthesis was performed using QuantiTect Reverse Transcription Kit (QIAGEN GmbH, Germany). cDNAs of interest were quantified using 0.1 µg of total cDNA using a StepOne Real-Time PCR System (Thermo Scientific) and TaqMan assay-specific primers (Supplementary Table 1) and master mix (Thermo Scientific). As endogenous control, glyceraldehyde 3-phosphate dehydrogenase (GAPDH) was used. StepOne Software (version 2.0.2) was used for evaluation.

For determining seRNA-plasmid concentrations after in vivo application, whole DNA was isolated from different mouse tissues using QIAamp DNA Mini Kit (QIAGEN, Germany) according to manufacturer instructions. Plasmid concentrations were determined in 0.1 µg of total DNA per tissue by qPCR. For quantification, a calibration curve was generated for each seRNA plasmid based on qPCR Ct values. Results were corrected for non-specific primer binding by subtraction of untreated animal values from those of treated animals. Resulting Fig. 7b and c was created with BioRender.com.

### Statistical analysis and data evaluation

Data are given as mean (s.d.). Analysis of variance (ANOVA) was used for all in vitro comparisons. A *p*-value of ≤ 0.05 was considered as significant. Significance levels of 0.05, 0.01, and 0.001 were displayed with one to three stars.

### In vivo experiments

#### Tumor cell preparation

Human glioblastoma U87 cells were used for tumor cell implantation in mice when a confluency of 80% was reached. A cell suspension of 10^6^ U87 cells in 20 µl PBS was created and stored in a 1 ml Eppendorf tube on ice until use. Storage time was limited to a maximum of six hours.

### Surgery

30 min prior to surgery, mice received Carporfen 4 mg/kg subcutaneously (Rimadyl®, Zoetis). Anesthesia was induced by isoflurane inhalation. Using a microinjector (SMARTouch, UMP3, WPI) and a microsyringe (NanoFil 10 µl, WPI), 4 µl with 2 × 10^5^ U87 cells in total were injected into the striatum of the right hemisphere, at the stereotaxic coordinates 2.4 mm lateral, 0.5 mm rostral, and 4 mm ventral of bregma. The syringe was left in place for five minutes and then removed slowly (1 mm/2 min). Additional Carprofen was given for two more days after surgery. Daily scoring of the animals followed to quantify severity levels.

Two days after tumor cell injection, mice received microinjection of either the therapeutic caspase-expressing seRNA-construct (*n* = 10), the control construct expressing eGFP only (seRNA-eGFP, *n* = 8) or sodium chloride (NaCl, *n* = 2), using the same stereotaxic coordinates as described above. For intratumoral seRNA-construct injection preparation of plasmid loaded lipoplexes (Lipofectamine 3000) was performed^71^. In brief, 2.5 µg seRNA-encoding plasmid DNA was incubated with 2.5 µl of P3000 reagent at RT for 10 min. Afterward, 5 µl of Lipofectamine 3000 reagent was added and mixed by vortexing for 30 s. Then, the solution was incubated at RT for 30 min. Intratumorally microinjection of 4 µl (1 µg seRNA encoding construct) was conducted with a speed of 0.25 µl/min. The total number of indicated animals were treated in four independent experiments. seRNA-loaded lipoplexes were prepared freshly for each animal and used instantly.

#### Magnetic resonance imaging (MRI)

MRI was performed on treated mice on days three, seven, and fourteen after the transfer of seRNA or sodium chloride. MRI was performed on either a 9.4 Tesla scanner (BioSpec 94/20; Bruker BioSpi), using a 1H quadrature cryogenic cooled surface coil (CryoProbe, Bruker BioSpin) or a 3 Tesla MRI system (Achieva®, Philips Healthcare, Best) with an 8 Channel Volumetric Rat Array (Rapid Biomedical GmbH, Rimpar). Mice were anaesthetized with isoflurane, and vital parameters were controlled by a custom-made setup (Medres; Cologne). After an initial localizer sequence, T2-weighted images were obtained. On the 9.4 T Bruker scanner, we used the following parameters: imaging method = RARE, RARE-factor = 8, repetition time = 5500 ms, echo time = 10.833 ms, acquisition matrix = 256 × 256; slice thickness = 0.5 mm, and number of averages = 2. For the 3 Tesla scanners, we worked with the following parameters: Slice thickness 0.5 mm, fast imaging mode = TSE, TSE factor = 5, acquisition matrix = 132 × 130, echo time = 37 ms, repetition time = 2794 ms. With both MRI systems, 28 slices were scanned.

#### Histology

Mice were sacrificed by decapitation after the last MRI. Brains were harvested and stored at − 80 °C until further analyses. Brains were cut coronally in 20 µm thick slices (Leica CM3050). To investigate proliferation, tissue was stained against Ki67 (1:500, Rabbit Polyclonal, ab15580, Abcam), using a fluorescent secondary antibody (1:500, Alexa- Fluor Goat anti- Rabbit 568 nm IgG (H + L), Invitrogen, Thermo Fisher). All nuclei were counter-stained with Hoechst (1:500, Hoechst 33342, Life Technologie). Images were analyzed with an inverted fluorescent microscope in phase contrast (Keyence BZ-9000E, Keyence), and representative pictures were acquired.

### Image Analysis

Tumor volumes were quantified in vivo on MR images using the software program VINCI 5.06 (Max Planck Institute for Neurological Research Cologne)^72^. To define tumor volumes, volumes of interest (VOIs) were defined in each animal and at every investigated time point.

### Statistics & reproducibility

Calculations were performed using the software GraphPad Prism (version 9.1.2). Normal distribution was analyzed via D’Agostino and Pearson test. Group differences were assessed by one-way repeated measures analysis of variance (ANOVA) and performed in both groups for the factor time. In addition, Greenhouse Geisser correction was conducted to control for violations of sphericity. Statistical significance was assumed at *p* < 5%. Results are shown in a box and whiskers plot, reporting the median, quartiles, and extreme values as well as single values for every time point. No statistical method was used to predetermine sample size, and no data were excluded from the analyses. The investigators were not blinded to allocation during experiments and outcome assessment. Each experiment was performed in at least three independent approaches. Representative micrographs were also examined in three or more independent biological replicates.

### Reporting summary

Further information on research design is available in the Nature Portfolio Reporting Summary linked to this article.

## Supplementary information

Supplementary Information

Description of Additional Supplementary Information

Reporting Summary

## Source data

Source Data

## Data Availability

All data that support the findings of this study are provided in the source data file and are additionally available from the corresponding author without restrictions upon request within 10 business days. All primer information is provided in the supplementary information files. The nanopore sequencing data have been deposited to the NCBI Gene Expression Omnibus (GEO) with the dataset identifier GSE283402. Source data are provided in this paper.

## Code availability

All computer codes used to analyze the data are indicated in this published article and are provided as Supplementary Code 1 and Supplementary Code 2.
