# Supplementary material for: Selectively expressed RNA molecules as a versatile tool for functionalized cell targeting
Source: Nat Commun. 2025 Jan 6;16:420. doi: 10.1038/s41467-024-55547-6 (PMC11704337; doi:10.1038/s41467-024-55547-6)
Supplement: Supplementary file 1 — Supplementary Information [file 41467_2024_55547_MOESM1_ESM.pdf]

Supplementary Figure 1. Keratin expression analysis

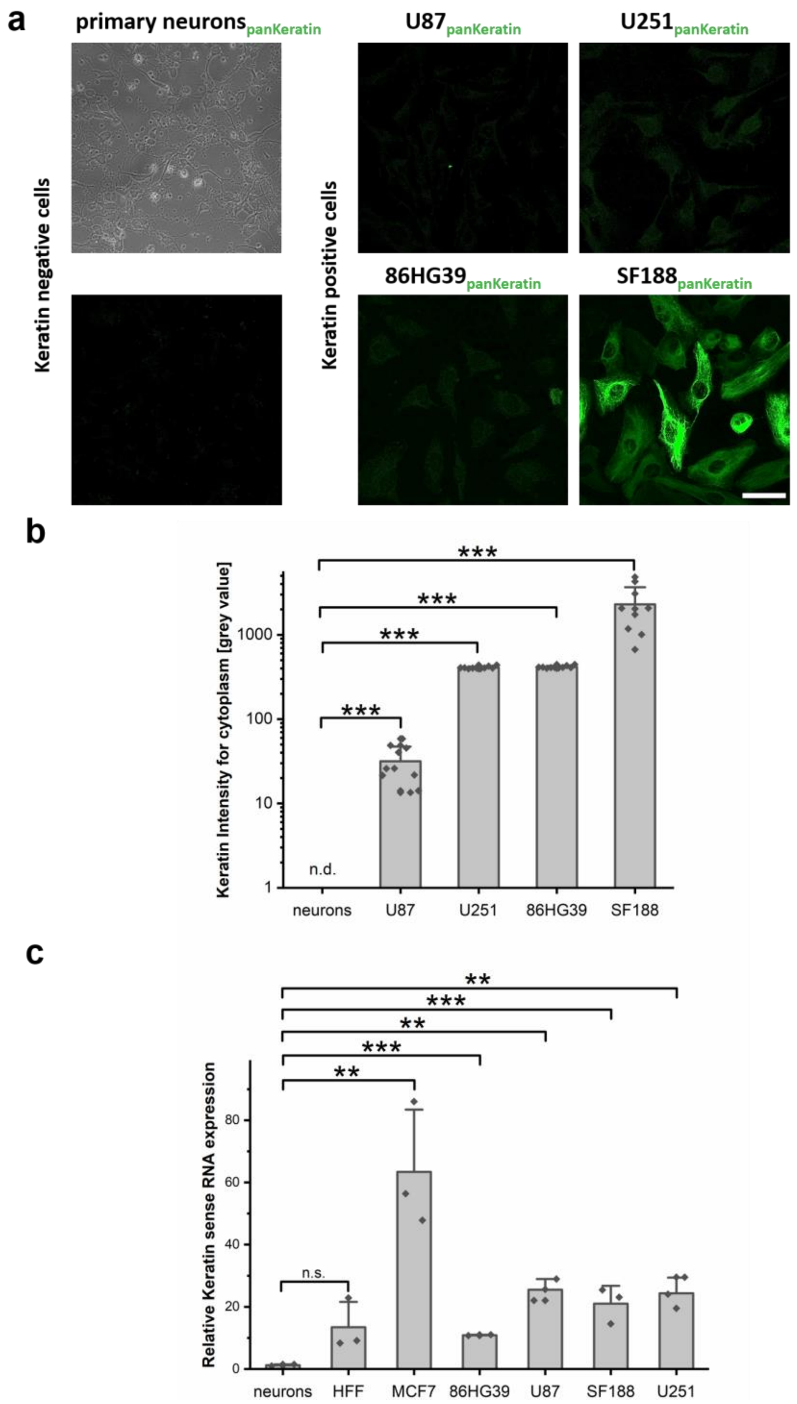

Keratin 13 expression analysis on protein and RNA level. (a) Indicated cell lines were stained for keratin. Primary neurons were used as cell type lacking keratin. After cultivation for 24 h, cells were stained as already described. All fluorescent images were taken by confocal microscopy using appropriate filter settings and 40x, EC Plan Neofluar/Ph3 (Carl Zeiss). For cell visibility, neurons are also shown in phase contrast. For all micrographs, settings were kept the same. Scale bar = 50 µm. (b) Keratin fluorescence intensities were analyzed using python as program: Cytoplasmic fluorescence intensities were measured and are given as mean grey

values per image with s.d. 10 images from three independent stainings were analyzed for each cell line. (c) Keratin sense RNA levels were quantified by qRT-PCR, using primers recognizing the sense-antisense interaction region. Equal total RNA amounts were analyzed for each cell line. Results were normalized relative to pr. neurons and are given with s.d. n = 3 independent experiments.

Supplementary Figure 2. Target cell specific seRNA activation upon use of shortened antisense domains

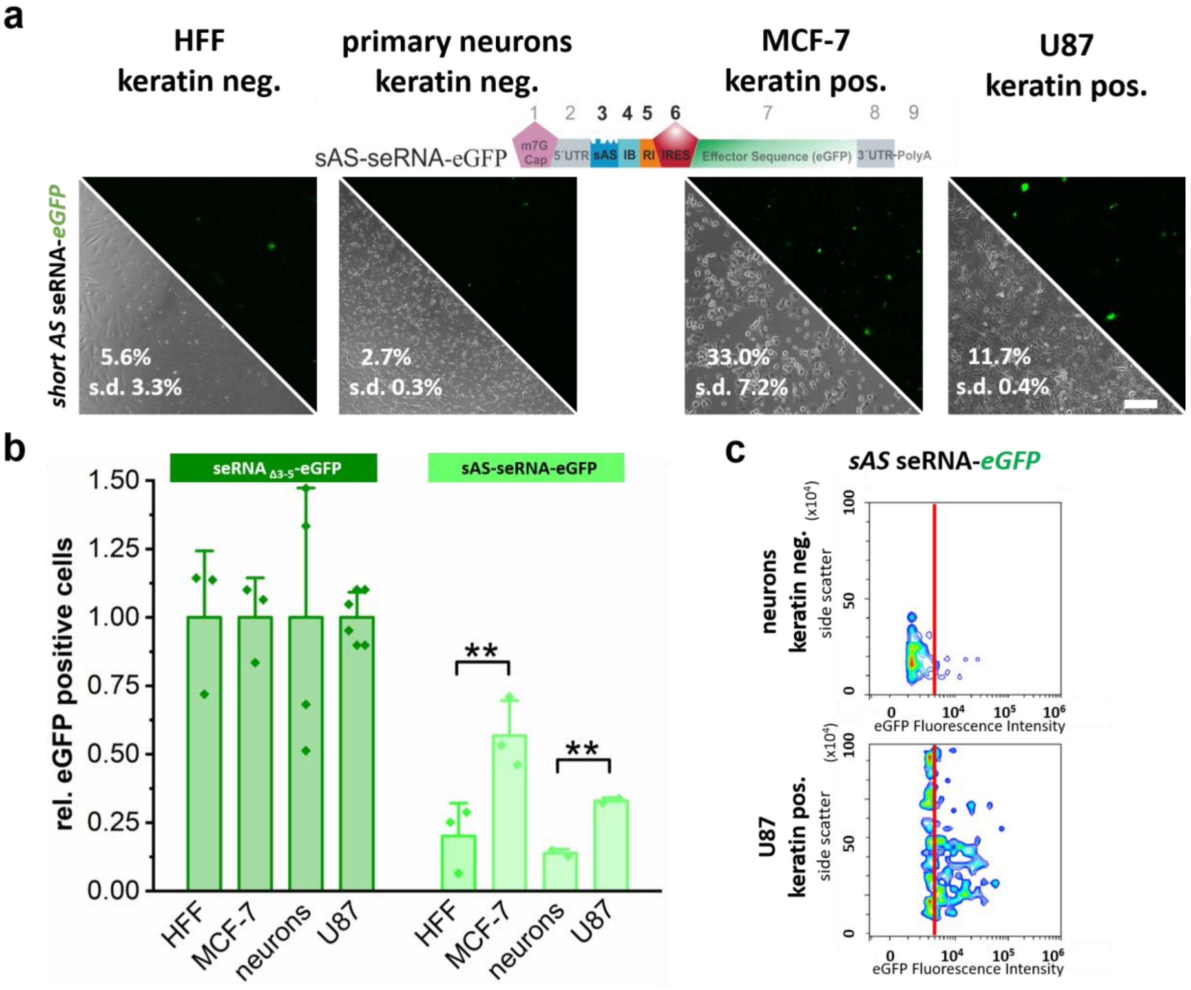

Antisense domain of construct seRNA-eGFP with specific targeting of keratin sense RNA was shortened from 625 to 80 nucleotides to result in construct sAS-seRNA-eGFP. This construct was transferred into non-target (HFF and primary neurons) and target cancer cells (MCF-7 and U87). GFP expression upon activation was visualized by microscopy and quantified by flow cytometry (indicated percentage) (a). Values were compared to the constitutively active seRNA-GFP$_{\Delta 3\text{-}5}$ construct (see figure 2) and are also given as relative values (b). Flow cytometry based eGFP intensity blots indicate specific effector expression in target cells but also show reduced intensity values compared to long antisense domain constructs.

Supplementary Figure 3. Repetitive transfection efficiency of seRNAs

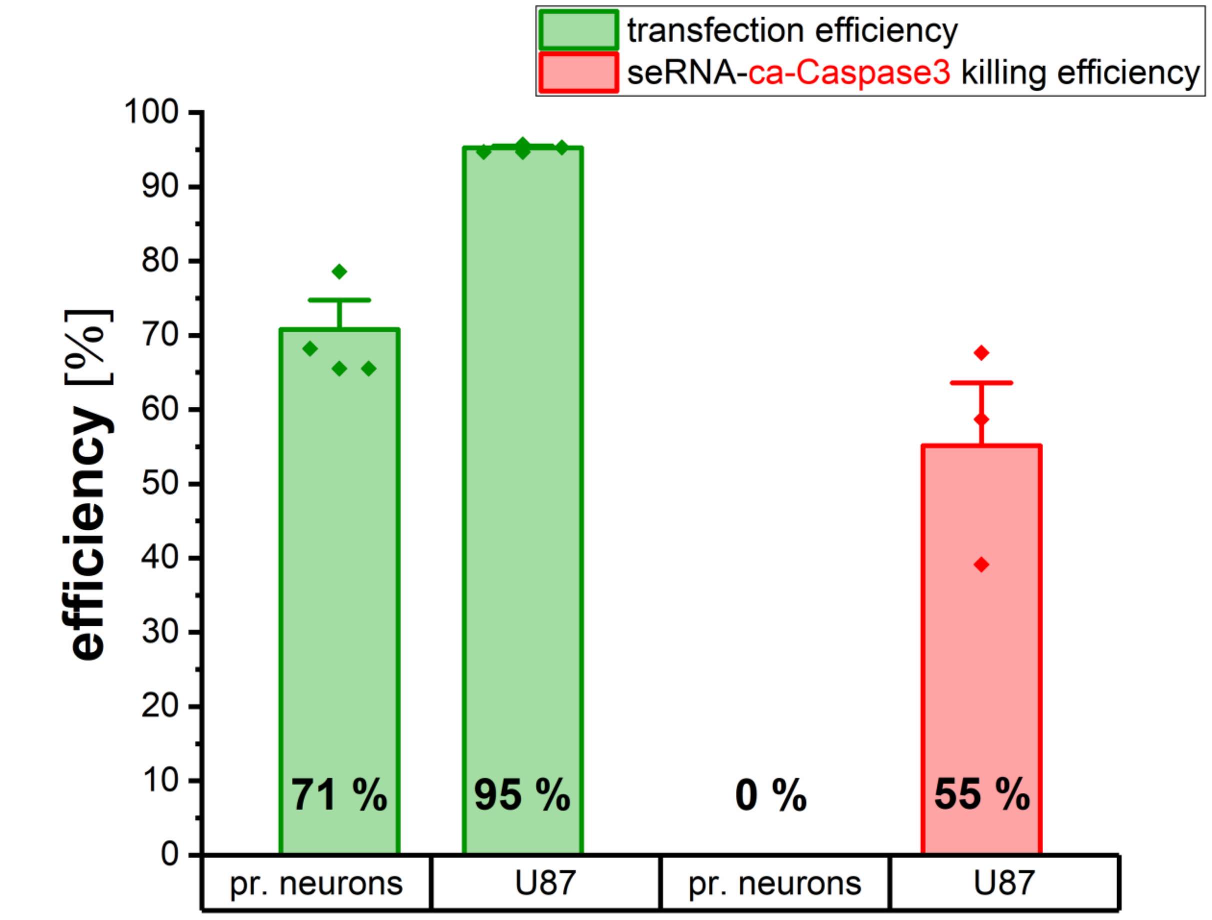

Dual transfer at 24 h intervals of eGFP plasmid and seRNA-ca-Caspase3 into U87 glioblastoma (target) and embryonic rat cortical neurons (non-target) cells with subsequent flow cytometry analysis 24 h after second transfer. Both cell types were characterized by high transfection efficiencies using lipofectamine 3000 as transfection reagent. However, seRNA activation with subsequent caspase activation occurred only in target cells. Data argue for enhanced seRNA efficacy upon repetitive treatment in target cells while healthy tissue remains unaffected. n = at least 3 independent experiments.

Supplementary Figure 4. Transfection efficiency control and seRNA specificity

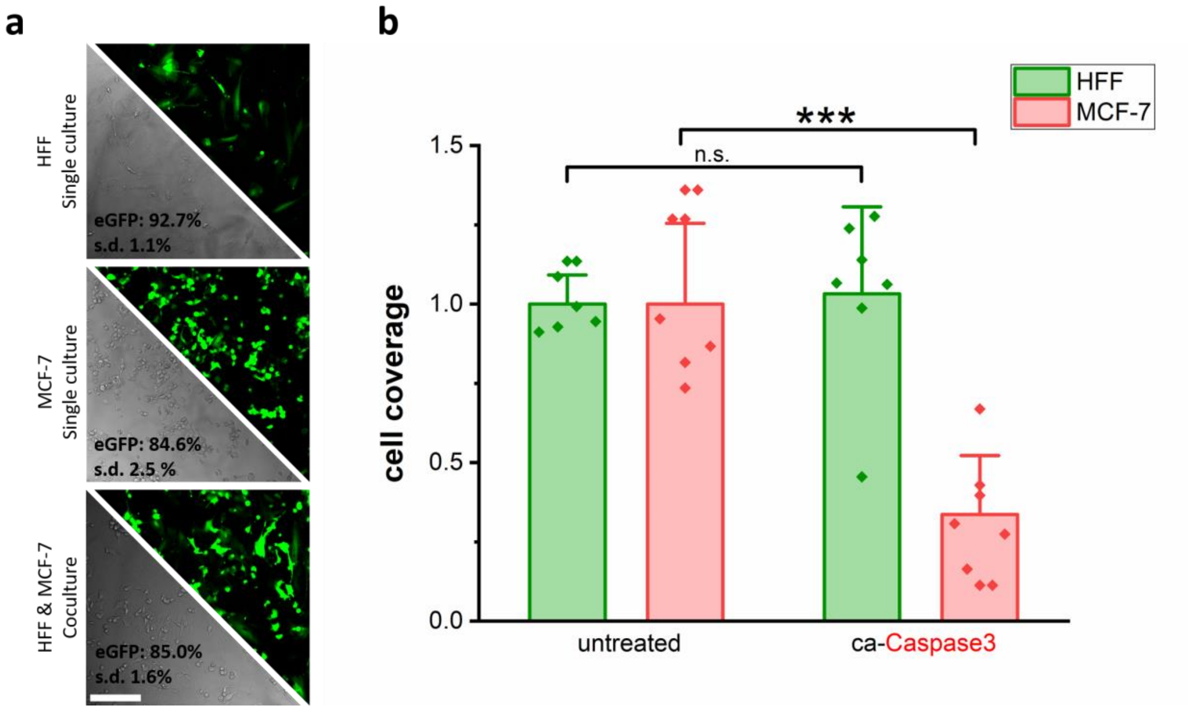

To confirm high and comparable transfection efficiencies for all cells used in Fig. 4g, indicated cell types were transfected separately and as co-culture using IVT eGFP-mRNA. Growth conditions were kept the same for all cultures. Transfection efficiencies are indicated based on flow cytometry analyses. Scale bar = 200 µm (a). Co-cultures of stably GFP-expressing HFF (non-target) and red nuclear labeled MCF-7 target cells were transfected with seRNA-ca-Caspase3 as indicated in Fig. 4. 24 h after transfection cell viability was quantified based on fluorescent green/red coverage of confocal images from 3 independent experiments. Note the strong reduction of target cells, while non-target cells remained fully unaffected (b).

Supplementary Figure 5. Glioblastoma cell lines in co-culture with rat primary neurons

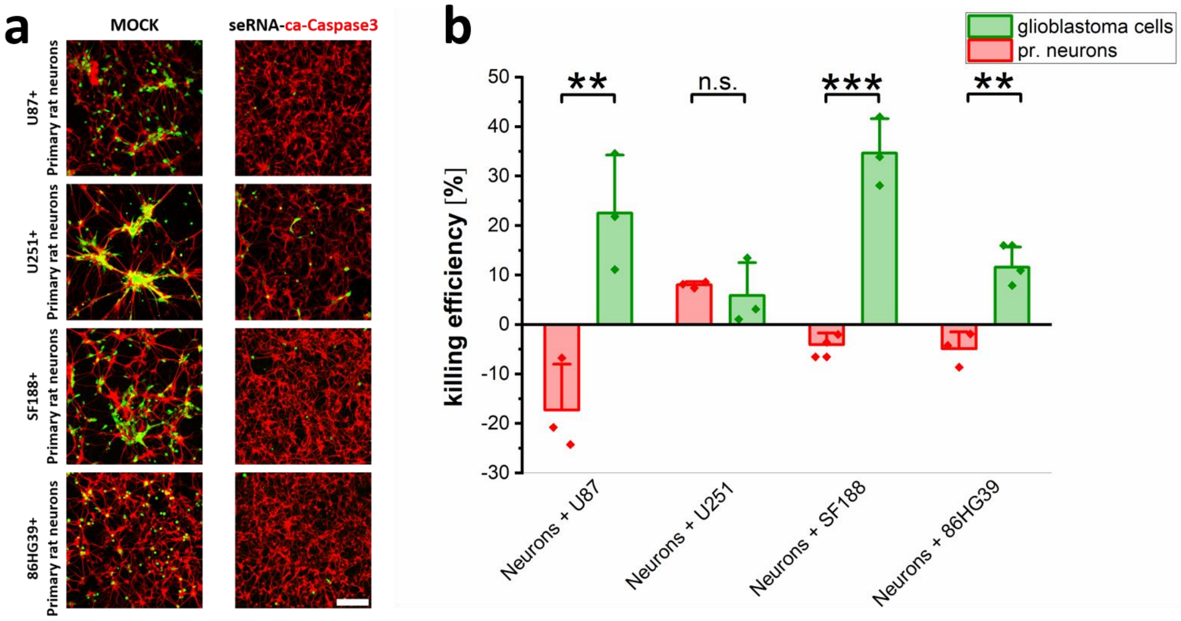

Treatment of co-cultures of primary rat cortical neurons and glioblastoma cell lines. (A) Stably cytoplasmic eGFP expressing glioblastoma cells were generated by transduction using lentiviral particles expressing GFP with Neomycin antibiotic marker (Amsbio) with a MOI of 1 according to the manufacturer protocol. We selected with different concentrations of G418 (Sigma Aldrich) diluted in media (86Hg39 800 µg/mL, U251 800 µg/mL, SF188 850 µg/mL and U87 850 µg/mL). 3 x 10^5 primary rat neurons were seeded on 0,01% PLL-coated substrates. After 24 h of incubation 3 x 10^4 glioblastoma cells were added. We used NB Media for all cell types and co-cultures (see Material and Methods). 24 h later co-cultures were transfected with 2 µg seRNA-ca-Caspase3 using lipofectamine 3000 (ThermoFisher, USA) or a MOCK RNA as control (Xenopus elongation factor 1α gene RNA). After additional 21 hours of incubation and 3 h before microscopy, microtubules of living cells were stained with SiR-tubulin (Spirochrome, 1:1000 dilution, red). Control cells remained untransfected but were cultured and stained the same way. Scale bar = 200 µm. (B) To quantify the killing efficiencies for glioblastoma target cells and neurons as non-target cells, we analyzed the co-cultures by flow cytometry. We compared the cell numbers of the MOCK treated with the seRNA-ca-Caspase3 treated samples; the change in percent in cell number is given as killing efficiency. Note that only U251 and primary neurons were characterized by spherical cell cluster formation when incubated in co-culture. Such growth behavior might be the reason for reduced U251 killing efficiency in co-culture only and some loss of primary neurons after seRNA treatment. n = 3 independent experiments.

Supplementary Figure 6. Influence of RNase H on seRNA activation

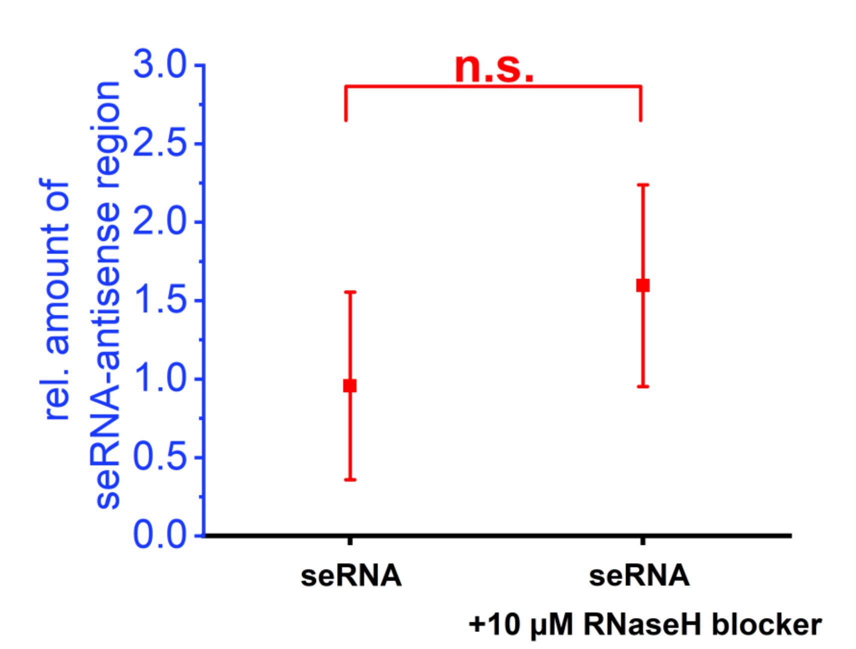

For RNase H inhibition, U87 cells were incubated with 10 µM of (Z)-5-(3,4-dihydroxybenzylidene)-3-(4-hydroxyphenyl)thiazolidine-2,4-dione (Life Chemicals, Canada) in the medium for 3 h. After washing, cells were transfected with appropriate seRNA plasmids and incubated for additional 8 hours before crude RNA isolation. Upon blocking RNase H activity, we enhanced concentration of undigested, full-length seRNA and therefore slightly increased amounts of 5'regions in two-probe qRT-PCR analyses. Data therefore confirm RNase H partial influence in recognition and digestion of formed dsRNA domains upon expression of seRNAs in target cells. n = 4 independent experiments.

Supplementary Figure 7. High resolution micrographs of all used cells

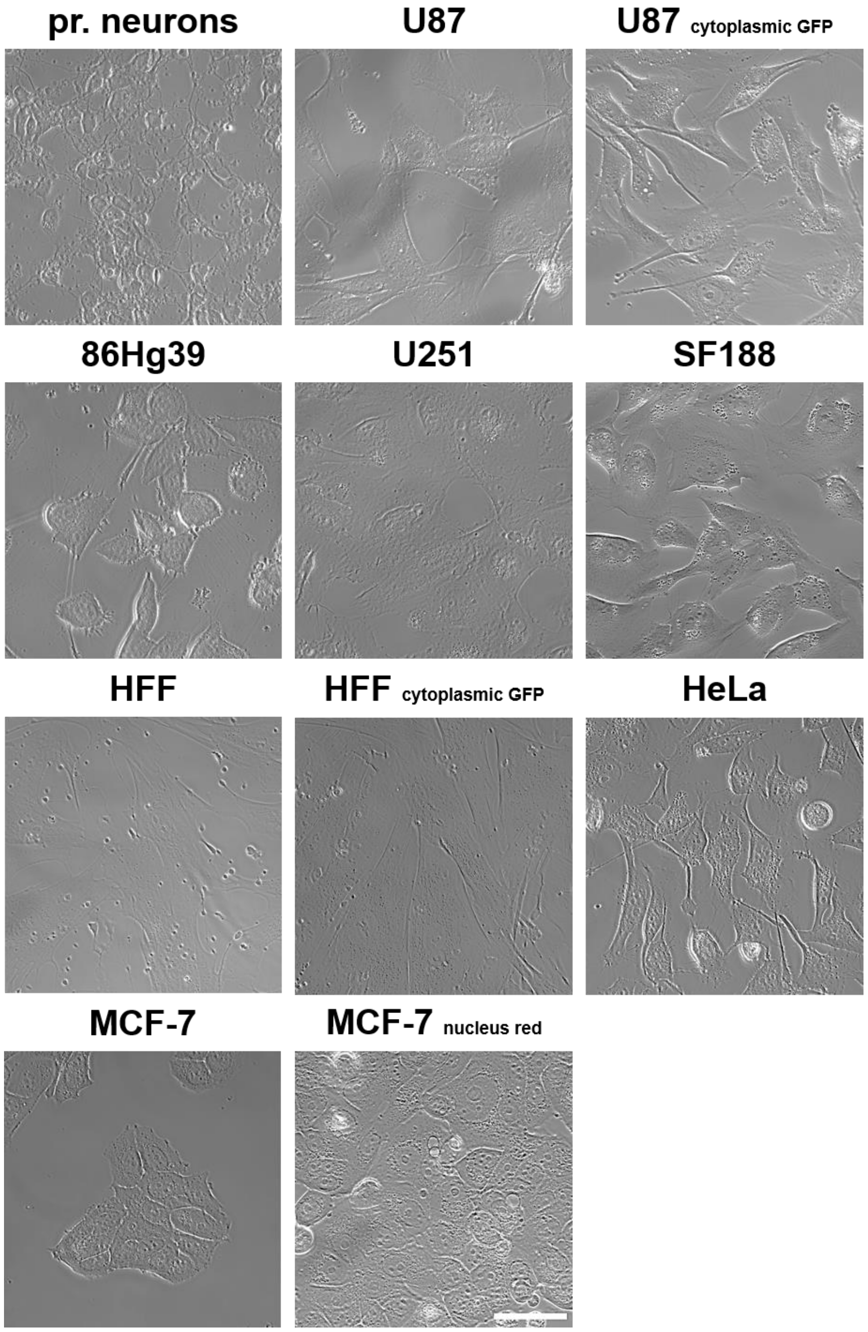

All cell types used in this paper were morphologically verified based on microscopy. After cultivation for 24 h, cells were washed once with PBS for 5 min at 37 °C. All images were taken by confocal microscopy using appropriate filter settings and a Plan-Apochromat 40x/1.3 Ph3 objective (Zeiss) for phase contrast imaging. For all images, settings were kept identical. We made sure, that all single-cell type and co-culture experiments were performed with cell morphologies as indicated here. Scale bar = 50 µm.

Supplementary Figure 8. Gating Strategy

a

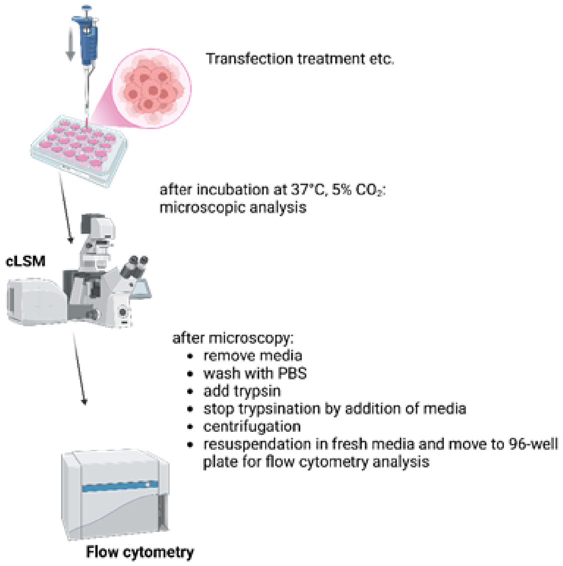

b ➤ Example for an eGFP construct in U87 target cells

1.

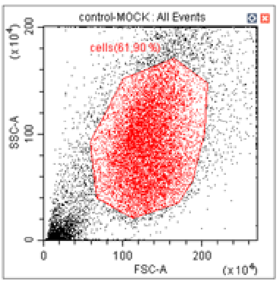

First gate includes all living cells from MOCK-control or untreated cells

2.

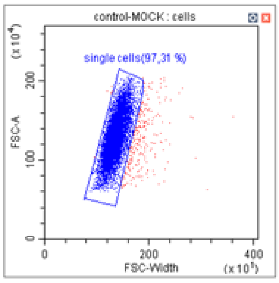

Second gate includes all single cells -> not essentially if properly resuspended and appropriate flow rate applied

3.

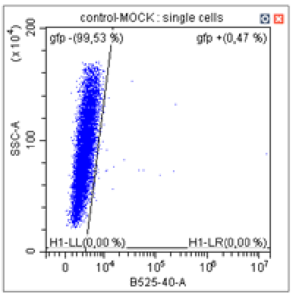

Third gate divides GFP negative from GFP positive cells

4.

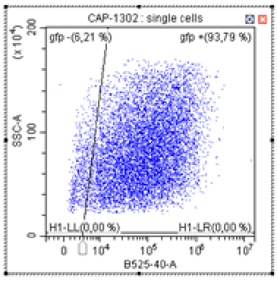

Representative blot of an eGFP positive control with this gating strategy. eGFP positive cells are evaluated in percent, together with the microscopic images

Supplementary Figure 8a was created in BioRender. Hoffmann, B. (2024) https://BioRender.com/s08i075.

Supplementary table 1

a) **Primer for seRNA construction**	**Sequence**	**Restriction enzymes**
**P**1 hK13 antisense fw	*CCGACCGGTTGATGTCGGCCTCCACGCTCT*	AgeI
**P**2 hK13 antisense rev	*CCGGAATTCATGAGCCTCCGCCTGCAGAG*	EcoRI
**P**3 IRES blocker 1 fw	*CCGGAATTCGTTATGTTATGAACTGCTTCCTTCACGACATTCAACAGACCTTGTTATGTTATTAAAAGTCAGGTCGGATCAAGCCAT*	EcoRI
**P**4 IRES blocker 2 fw	*CCGGAATTCGTTATGTTATAAGCTTCCAGAGGAACTGCTTGTTATGTTATTAAAAGTCAGGTCGGATCAAGCCAT*	EcoRI
**P**5 IRES blocker 3 fw	*CCGGAATTCGTTATGTTATAGATCAGATCCCATACAATGGGGTACCTTCTGTTATGTTATTAAAAGTCAGGTCGGATCAAGCCAT*	EcoRI
**P**6 IRES blocker 1-3 rev	*CTAGTCTAGACATGGTTGTGGCCATATTATCATCGTGTTTTTCAAAGGAAAACCACGT*	XbaI
**P**7 RNase Inhibitor region part 1 fw	*GAATTCGTTATGTTATGACCTTGCTCGTCAAGAAGACAGTTATGTTATTAAAAGTCAGGTCGGATCAAGCCATAGTACGGAAAAAACTATGCTACCTGTGAGCCCCGTCCAAGGACGTGC*	EcoRI
**P**8 RNase Inhibitor region part 1 rev	*GGCCGCACGTCCTTGGACGGGGCTCACAGGTAGCATAGTTTTTTCCGTACTATGGCTTGATCCGACCTGACTTTTAATAACATAACTGTCTTCTTGACGAGCAAGGTCATAACATAACG*	
**P**9 RNase Inhibitor region part 2 fw	*GGCCGCTAAAAGAAGTCAGGCCATCACAAATGCCACAGCTTGAGTAAACTGTGCAGCCTGTAGCTCCACCTGAGAAGGTGTAAAAAAGTTATGTTATG*	
**P**10 RNase Inhibitor region part 2 rev	*GGATCCATAACATAACTTTTTTACACCTTCTCAGGTGGAGCTACAGGCTGCACAGTTTACTCAAGCTGTGGCATTTGTGATGGCCTGACTTCTTTTAGC*	BamHI
**P**11 EMCV IRES fw	*CGCGGATCCCCCCCCTAACGTTACTGGCCGAAGCCG*	BamHI
**P**12 EMCV IRES rev	*CTAGTCTAGACATGGTTGTGGCCATATTATCATCGTGTTTTTCAAAGGAAAACCACGT*	XbaI
b) **HBx specific antisense exchange**	nucleotides 1424 to 2053 of HBV genome	AgeI, EcoRI
c) **Probes for qRT-PCR**	**Target gene**	**Species**
	eGFP, Mr04097229_mr, Thermo Fisher, USA	non
Custom made	KLI-IB1 (quantification of 5´seRNA region)	non

	GAPDH, Hs02786624_g1, Thermo Fisher, USA	human
	GAPDH, Rn01775763_g1, Thermo Fisher USA	rodent
	GAPDH, Mm99999915_g1, Thermo Fisher USA	murin
	IL6, Hs00174131, Thermo Fisher, USA	human
	IL6, Rn01410330, Thermo Fisher, USA	rodent
	TNFα, Hs00174128, Thermo Fisher, USA	human
	TNFα, Rn9999901, Thermo Fisher, USA	rodent
	CASP3, Hs00234387_m1, Thermo Fisher, USA	human
Target Ker RNA fw	ATGAGCCTCCGCCTGCAGA	
Target Ker RNA rev	TGATGTCGGCCTCCACGCT	
**d) Plasmids used for co-transfection**	**Plasmid**	**Distributor**
GFP HBx	pGFP-HBx NESM (Plasmid #24932)	Addgene
HBx	HBx (Plasmid #65463)	Addgene
MOCK	pSecTag-HygroB	Invitrogen

Supplementary table 2. Length information for seRNA domains and full-length constructs.

Domain	description	construct	Length (bp)
3	Antisense	keratin	625
		short keratin	80
		HBx	629
4	IRES-Blocker	IB 1	33
		IB 2	22
		IB 3	23
5	RNase-Inhibitor	part 1	70
		part 2	77
6	EMCV IRES		574
7	Effector	eGFP	720
		ca-caspase3	873
Full length 1-9	seRNA$_{\Delta 3\text{-}5}$-eGFP		2029
	AS-seRNA-eGFP		2157
	sAS-seRNA-eGFP		1612
	seRNA$_{\Delta 3\text{-}5}$-ca-caspase3		2169
	AS-seRNA-ca-caspase3		2309
	seRNA-HBx-ca-caspase3		2312
