# Supplementary material for: Selectively expressed RNA molecules as a versatile tool for functionalized cell targeting
Source: Nat Commun. 2025 Jan 6;16:420. doi: 10.1038/s41467-024-55547-6 (PMC11704337; doi:10.1038/s41467-024-55547-6)
Supplement: Supplementary file 2 — Description of Additional Supplementary Information [file 41467_2024_55547_MOESM2_ESM.docx]

Supplementary Code 1:

Python based software tool for the analysis of cell coverage based on differently fluorescently labeled cells within an image. Both fluorescence channels (green and red) were analyzed separately. A binary mask was created from each image using a preselected gray value threshold (1000). Subsequent processing using morphological closing followed by morphological opening allowed coverage calculation.

Supplementary Code 2:

Python based software code for intensity calculations of fluorescently labeled cells. Three channel confocal images of cells (two fluorescent channels for antibody labeled keratin and DAPI nuclear staining as well as one wide field channel) were taken.

To identify in a first step large cell free areas in each image, the wide field channel was used and a contrast limited adaptive histogram equalization (CLAHE) was performed followed by a local gradient filter with a disk shaped structure element (radius of 17 pixels). Small objects were additionally removed and small holes were filled to result in a substrate free mask.

To same wide filed images, a local Otsu filter was applied to create a binary mask of cell areas. Following a logical AND of the binary mask of cell areas with the substrate free mask, the binary mask of cell areas was further refined.

Images of the Dapi channel were smoothed using a Gaussian filter (sigma=3.0) and converted into a binary mask using triangular thresholding.

Finally, refined binary cell area mask and binary nuclear mask were combined and used in the keratin fluorescence channel to determine the exact intracellular fluorescence intensity.
