# Supplementary material for: Selectively expressed RNA molecules as a versatile tool for functionalized cell targeting
Source: Nat Commun. 2025 Jan 6;16:420. doi: 10.1038/s41467-024-55547-6 (PMC11704337; doi:10.1038/s41467-024-55547-6)
Supplement: Supplementary file 3 — Reporting Summary [file 41467_2024_55547_MOESM3_ESM.pdf]

nature portfolio

Corresponding author(s): Dr. Bernd Hoffmann

Last updated by author(s): Dec 11, 2024

Reporting Summary

Nature Portfolio wishes to improve the reproducibility of the work that we publish. This form provides structure for consistency and transparency in reporting. For further information on Nature Portfolio policies, see our Editorial Policies and the Editorial Policy Checklist.

Statistics

For all statistical analyses, confirm that the following items are present in the figure legend, table legend, main text, or Methods section.

n/a	Confirmed	
☐	☒	The exact sample size (n) for each experimental group/condition, given as a discrete number and unit of measurement
☐	☒	A statement on whether measurements were taken from distinct samples or whether the same sample was measured repeatedly
☐	☒	The statistical test(s) used AND whether they are one- or two-sided<br>*Only common tests should be described solely by name; describe more complex techniques in the Methods section.*
☐	☒	A description of all covariates tested
☒	☐	A description of any assumptions or corrections, such as tests of normality and adjustment for multiple comparisons
☐	☒	A full description of the statistical parameters including central tendency (e.g. means) or other basic estimates (e.g. regression coefficient) AND variation (e.g. standard deviation) or associated estimates of uncertainty (e.g. confidence intervals)
☐	☒	For null hypothesis testing, the test statistic (e.g. F, t, r) with confidence intervals, effect sizes, degrees of freedom and P value noted<br>*Give P values as exact values whenever suitable.*
☒	☐	For Bayesian analysis, information on the choice of priors and Markov chain Monte Carlo settings
☒	☐	For hierarchical and complex designs, identification of the appropriate level for tests and full reporting of outcomes
☒	☐	Estimates of effect sizes (e.g. Cohen's d, Pearson's r), indicating how they were calculated

Our web collection on statistics for biologists contains articles on many of the points above.

Software and code

Policy information about availability of computer code

Data collection	Cyt Expert 2.3.0.84, Beckmann Coulter for Flow cytometry data;<br>StepOne Software (version 2.0.2), Thermo Fisher for qRT-PCR experiments<br>LSM 710 Software ZEN 2.3 SP1 black (64 bit) for Microscopy images<br>Minion Mk1B sequencer (Oxford Nanopore Technologies)
Data analysis	GraphPad Prism (version 9.1.2) for statistical analyses;<br>Origin Pro 2019, Origin Lab Corporation<br>MinKnow acquisition software (Oxford Nanopore Technologies) v. 23.07.8<br>Customs codes for evaluations are indicated in the manuscript and provided as stand-alone versions as Supplementary Code 1 and Supplementary Code 2

For manuscripts utilizing custom algorithms or software that are central to the research but not yet described in published literature, software must be made available to editors and reviewers. We strongly encourage code deposition in a community repository (e.g. GitHub). See the Nature Portfolio guidelines for submitting code & software for further information.

Data

Policy information about availability of data

All manuscripts must include a data availability statement. This statement should provide the following information, where applicable:

- Accession codes, unique identifiers, or web links for publicly available datasets
- A description of any restrictions on data availability
- For clinical datasets or third party data, please ensure that the statement adheres to our policy

All data that support the findings of this study are provided in the source data file and are additionally available from the corresponding author without restrictions upon request within 10 business days. All primer information is provided in the supplementary information files. The nanopore sequencing data have been deposited to the NCBI Gene Expression Omnibus (GEO) with the dataset identifier GSE283402 [https://www.ncbi.nlm.nih.gov/geo/query/acc.cgi?acc=GSE283402].

Research involving human participants, their data, or biological material

Policy information about studies with human participants or human data. See also policy information about sex, gender (identity/presentation), and sexual orientation and race, ethnicity and racism.

Reporting on sex and gender	*Use the terms sex (biological attribute) and gender (shaped by social and cultural circumstances) carefully in order to avoid confusing both terms. Indicate if findings apply to only one sex or gender; describe whether sex and gender were considered in study design; whether sex and/or gender was determined based on self-reporting or assigned and methods used. Provide in the source data disaggregated sex and gender data, where this information has been collected, and if consent has been obtained for sharing of individual-level data; provide overall numbers in this Reporting Summary. Please state if this information has not been collected. Report sex- and gender-based analyses where performed, justify reasons for lack of sex- and gender-based analysis.*
Reporting on race, ethnicity, or other socially relevant groupings	*Please specify the socially constructed or socially relevant categorization variable(s) used in your manuscript and explain why they were used. Please note that such variables should not be used as proxies for other socially constructed/relevant variables (for example, race or ethnicity should not be used as a proxy for socioeconomic status). Provide clear definitions of the relevant terms used, how they were provided (by the participants/respondents, the researchers, or third parties), and the method(s) used to classify people into the different categories (e.g. self-report, census or administrative data, social media data, etc.) Please provide details about how you controlled for confounding variables in your analyses.*
Population characteristics	*Describe the covariate-relevant population characteristics of the human research participants (e.g. age, genotypic information, past and current diagnosis and treatment categories). If you filled out the behavioural & social sciences study design questions and have nothing to add here, write "See above."*
Recruitment	*Describe how participants were recruited. Outline any potential self-selection bias or other biases that may be present and how these are likely to impact results.*
Ethics oversight	*Identify the organization(s) that approved the study protocol.*

Note that full information on the approval of the study protocol must also be provided in the manuscript.

Field-specific reporting

Please select the one below that is the best fit for your research. If you are not sure, read the appropriate sections before making your selection.

- [x] Life sciences
- [ ] Behavioural & social sciences
- [ ] Ecological, evolutionary & environmental sciences

For a reference copy of the document with all sections, see nature.com/documents/nr-reporting-summary-flat.pdf

Life sciences study design

All studies must disclose on these points even when the disclosure is negative.

Sample size	No a priory sample size calculations were performed. Based on subsequent statistical analysis and G Power analysis, samples size were sufficiently large for all results indicated in the study.
Data exclusions	No experimental data were excluded
Replication	All experiments were repeated fully independently at least 3 times. Each experiment was analyzed independently and as sum
Randomization	Experimental design did not involve randomization
Blinding	For in vitro experiments no blinding was applied during data analysis. MRI data from mice glioblastoma were analyzed in blinded form. Furthermore, results were confirmed by a second scientist for randomly chosen data sets.

Reporting for specific materials, systems and methods

We require information from authors about some types of materials, experimental systems and methods used in many studies. Here, indicate whether each material, system or method listed is relevant to your study. If you are not sure if a list item applies to your research, read the appropriate section before selecting a response.

Materials & experimental systems

n/a	Involved in the study
☐	☒ Antibodies
☐	☒ Eukaryotic cell lines
☒	☐ Palaeontology and archaeology
☐	☒ Animals and other organisms
☒	☐ Clinical data
☒	☐ Dual use research of concern
☒	☐ Plants

Methods

n/a	Involved in the study
☒	☐ ChIP-seq
☐	☒ Flow cytometry
☐	☒ MRI-based neuroimaging

Antibodies

Antibodies used	Pan-Keratin antibody (Mouse mAb conjugated with Alexa Fluor® 488, C11, mAB#4545) (Cell Signaling Technology, USA); Ki67 primary antibody (1:500, Rabbit Polyclonal, ab15580, Abcam); secondary antibody (1:500, Alexa- Fluor Goat anti- Rabbit 568nm IgG (H+L), Invitrogen, ThermoFisher); nuclei counter-staining with Hoechst (1:500, Hoechst 33342, Life Technologie)
Validation	All antibodies are explicitly described and validated by the manufacturers

Eukaryotic cell lines

Policy information about cell lines and Sex and Gender in Research

Cell line source(s)	HFF-1 (order number SCRC-1041), MCF-7 (HTB-22), HeLa (CCL-2), U-87MG (HTB-14) , HEK293T (CRL-3216) all from ATCC<br>U-251 MG (9063001), SF188 (SCC282) all from Sigma-Aldrich
Authentication	Cell lines used had been purchased by commercial suppliers. No additional authentication was done
Mycoplasma contamination	All cell lines used were free of mycoplasma
Commonly misidentified lines (See ICLAC register)	No commonly misidentified cell lines had been used

Animals and other research organisms

Policy information about studies involving animals; ARRIVE guidelines recommended for reporting animal research, and Sex and Gender in Research

Laboratory animals	Mutant immunodeficient mice, MRI-Foxn1nu/nu; male, Charles River Laboratories, France
Wild animals	Study did not involve wild animals
Reporting on sex	All animals used in the study were male. At this moment we cannot state if results will be the same for female animals
Field-collected samples	Study did not involve samples collected in the field
Ethics oversight	As part of the animal experiment approval by the German Government (Landesamt für Natur, Umwelt und Verbraucherschutz North Rhine-Westphalia; number 81-02.04.2019.A396) all analysis were also approved by the Ethics committee for animal testing.

Note that full information on the approval of the study protocol must also be provided in the manuscript.

Plants

Seed stocks

Report on the source of all seed stocks or other plant material used. If applicable, state the seed stock centre and catalogue number. If plant specimens were collected from the field, describe the collection location, date and sampling procedures.

Novel plant genotypes

Describe the methods by which all novel plant genotypes were produced. This includes those generated by transgenic approaches, gene editing, chemical/radiation-based mutagenesis and hybridization. For transgenic lines, describe the transformation method, the number of independent lines analyzed and the generation upon which experiments were performed. For gene-edited lines, describe the editor used, the endogenous sequence targeted for editing, the targeting guide RNA sequence (if applicable) and how the editor was applied.

Authentication

Describe any authentication procedures for each seed stock used or novel genotype generated. Describe any experiments used to assess the effect of a mutation and, where applicable, how potential secondary effects (e.g. second site T-DNA insertions, mosiacism, off-target gene editing) were examined.

Flow Cytometry

Plots

Confirm that:

☒ The axis labels state the marker and fluorochrome used (e.g. CD4-FITC).

☒ The axis scales are clearly visible. Include numbers along axes only for bottom left plot of group (a 'group' is an analysis of identical markers).

☒ All plots are contour plots with outliers or pseudocolor plots.

☒ A numerical value for number of cells or percentage (with statistics) is provided.

Methodology

Sample preparation

24 h after transfection, the cells were trypsinized (0.05% trypsin-EDTA solution, Sigma Aldrich, USA) and centrifuged at 300g. Without fixation, cells were analyzed directly after resuspension in 250 µl of the corresponding culture medium. At least 10,000 cells were analyzed for granularity and size by forward scattering for each cell type.

Instrument

CytoFLEX S Flow Cytometer, Beckmann Coulter

Software

Cyt Expert 2.3.0.84, Beckmann Coulter

Cell population abundance

Post-sort fractions have not been used in this study.

Gating strategy

For accurate determination of cell population as well as positive and negative cells, untreated control cells were acquired in the FSC and SSC channels in all experiments. Furthermore, these control cells were used to set the gates for the detection of eGFP positive cells and in untreated cells the number of eGFP positive cells was set to < 1%, ensuring high sensitivity and specificity. Analogous detection was performed after live-dead staining, using additional dead controls to validate the positive signals in advance and to adjust the gates accordingly for live (non-red) and dead cells (red).

☒ Tick this box to confirm that a figure exemplifying the gating strategy is provided in the Supplementary Information.

Magnetic resonance imaging

Experimental design

Design type

Anatomical imaging

Design specifications

T2 contrast: TR=5500ms, effective TE=43,3 ms

Behavioral performance measures

none

Acquisition

Imaging type(s)

structural

Field strength

9.4T

Sequence & imaging parameters

Pulse Sequence: SE, Type: RARE, RARE factor 8, TE=10,833ms, TR=5500ms, matrix 256x256, slice thickness=0.5 mm, 28 slices

Area of acquisition

whole brain

Diffusion MRI ☐ Used ☒ Not used

Preprocessing

Preprocessing software	n/a
Normalization	n/a
Normalization template	n/a
Noise and artifact removal	n/a
Volume censoring	*Define your software and/or method and criteria for volume censoring, and state the extent of such censoring.*

Statistical modeling & inference

Model type and settings	n/a
Effect(s) tested	n/a

Specify type of analysis: ☐ Whole brain ☐ ROI-based ☐ Both

Statistic type for inference (See Eklund et al. 2016)	n/a
Correction	n/a

Models & analysis

n/a	Involved in the study
☒	☐ Functional and/or effective connectivity
☒	☐ Graph analysis
☒	☐ Multivariate modeling or predictive analysis
